# Association of BCC Module Roll-Out in SHG meetings with changes in complementary feeding and dietary diversity among children (6–23 months)? Evidence from JEEViKA in Rural Bihar, India

**DOI:** 10.1371/journal.pone.0279724

**Published:** 2023-01-05

**Authors:** Sudipta Mondal, William Joe, Santosh Akhauri, Putul Thakur, Abhishek Kumar, Narottam Pradhan, Prasann Thatte, Rakesh Kumar Jha, Apolenarius Purty, Indrajit Chaudhuri

**Affiliations:** 1 Monitoring Learning and Evaluation, Project Concern International, Bihar, India; 2 Institute of Economic Growth, Delhi, India; 3 Central University of Gujarat, Gandhinagar, India; 4 JEEViKA, Bihar Rural Livelihood Promotion Society (BRLPS), Patna, India; Indian Institute of Technology Roorkee, INDIA

## Abstract

**Objectives:**

Child dietary diversity is very low across rural communities in Bihar. Based on the experience of behavior change communication (BCC) module roll out in self-help group (SHG) sessions in rural Bihar, this study aims to assess the impact of the intervention on child dietary diversity levels in the beneficiary groups.

**Methods:**

The study is based on a pre-post study design whereby child dietary diversity is examined for a sample of 300 children (6–23 months old from 60 village organizations) during both pre-intervention as well as post-intervention phase. The latter consists of two types of group viz. a) children whose mothers were directly exposed to BCC module in SHGs sessions and b) those who were non-participants but may have indirect exposure through spillovers of BCC activities. Econometric analysis including logistic regression as well as propensity score matching techniques are applied for estimating the changes in dietary diversity in the post-intervention phase.

**Results:**

During the pre-intervention phase, 19% of the children (6–23 months) had adequate dietary diversity (eating from at least 4 out of 7 different food groups) and this increased to 49% among the exposed group and to 28% among the non-exposed group in the post-intervention phase. The exposed group have an odds ratio of 3.81 (95% CI: 2.03, 7.15) for consuming diverse diet when compared to the pre-intervention group. The propensity score matching analysis finds a 33% average treatment effect on the treated (ATT) for the group participating in BCC sessions at SHG events.

**Conclusion:**

BCC roll out among SHG members is an effective mode to increase dietary diversity among infants and young children. The impact on child dietary diversity was significantly higher among mothers directly exposed to BCC modules. The BCC module also improved knowledge and awareness levels on complementary feeding and child dietary diversity.

## Introduction

Notwithstanding rapid economic growth since the 1990s, the developmental performance of India is weighed down by a worrisome state-of-affairs in the field of child health and nutrition. The National Family Health Survey (NFHS 2019–21) of India finds that 35.5%, 32.1% and 19.3% of the children (below five years) continue to suffer from stunting, underweight and wasting, respectively [1]. While these indicators have shown reductions since early 2000s but these improvements are offset by recent deteriorations in certain other nutrition-related indicators for children. The increase in the prevalence of anemia (58.6% in NFHS 2015–16 to 67.1% in NFHS 2019–21) as well as severe acute malnutrition (7.5% in NFHS 2015–16 to 7.7% in NFHS 2019–21) among children under-five is rather disturbing [1,2]. Such burden has a detrimental impact on the income and productivity levels of the concerned individuals as well as on the overall economic growth and welfare potential of the countries [3]. The stagnancy (and worsening) of some of these indicators also undermines the programmatic efforts to improve maternal and child nutrition in India and unequivocally calls for devising alternative strategies to address this long-standing problem in public health [4–7].

This paper contributes in this direction and aims to analyze the impact of Behavior Change and Communication (BCC) module roll out in SHG meetings on complementary feeding practices in rural Bihar. The findings add to the evidence base on the role of health and nutrition layering interventions in community settings (such as SHGs) with special reference to complementary feeding practices. It is hypothesized that exposure of SHG members to BCC modules should lead to improvements in complementary feeding practices and should have a spillover on the community (those unexposed to BCC module). Based on a cross-sectional pre- and post-intervention exposure design, the three specific objectives of the analysis are listed as follows: First, to examine the change in knowledge levels of mothers regarding complementary feeding and dietary diversity in the pre- and post-intervention setting. Second, to estimate the impact of the intervention on the level of dietary diversity and minimum acceptable diet among children 6–23 months. And finally, to understand the direct effect of exposure to the intervention among participants of SHG events vis-à-vis the indirect effect among the non-participants. The findings are expected to inform policymaking on BCC strategies for improving complementary feeding practices in rural geographies that impose severe time and resource constraints.

As such, since the advent of the Demographic and Health Surveys (DHS) in 1980s, a large number of studies have examined the drivers and determinants of child undernutrition among low- and middle-income countries including India [8–11]. Most of this evidence on social and biological determinants is translated into action by the national and international organizations including the union and the state governments in India through various flagship programmes that seek to accelerate reductions in child undernutrition globally. However, sub-optimal achievements of some of the nutrition-related programmes, such as the Integrated Child Development Services (ICDS), calls for a scrutiny and re-calibration of the policy matrix [12,13]. In this regard, particular interest has emerged in directly addressing—what is presumably—the most important determinant of nutrition i.e., complementary feeding practices. Several studies and policy guidelines now lay a clear emphasis on influencing dietary intake and dietary diversity among children in the age group of 6–23 months [14–18]. It is worth noting that adequate dietary intake among children (6–23 months) is among the slowest improving indicators in India [1]. For instance, the proportion of children (6–23 months) receiving an adequate diet in India was estimated to be 9.6% in NFHS 2015–16 and 11.3% in NFHS 2019–21. Therefore, attaining rapid improvements in complementary feeding practices is an important area for policymaking. In particular, greater focus is desired on the first 1000 days, including the 6–23 months window, with dietary counseling and behavior change being accorded highest priority for improving nutrition [19].

It is argued that a pro-active community outreach model can be an effective means to overcome the limitations of the health centre or ICDS centre based delivery approach to improve complementary feeding practices [20]. Some studies also recommend such outreach via community health workers but their effectiveness, acceptance, coverage and workload imbalances are fundamental constraints in leveraging their role toward improving complementary feeding practices [21–24]. Hence, development and testing of alternate strategies to address the problem of inter-personal counseling for nutrition assumes huge relevance in vastly rural and populous environments with overwhelming physical and financial barriers [25–28]. In this context, layering of health and nutrition interventions on rapidly expanding platform of women self-help group (SHG) networks across low-income geographies is viewed as a unique opportunity to influence complementary feeding norms and practices [29,30]. This is largely attributable to the attractiveness of the SHG model as a cost-effective option for governments and donors to quickly transmit behavior change messages and use the social capital of the groups to reinforce these norms through peer-support as well as sanctioning of any deviant practices [31–33].

Nevertheless, there is limited yet mixed evidence on the effectiveness and impact of such SHG centric interventions on complementary feeding practices [34–36]. For instance, Kushwaha et al (2014) finds that peer counselling by the mother support groups had a favorable impact on infant and young child feeding practices in Lalitpur district of Uttar Pradesh. Similarly, in the context of Ethiopia, Kang et al (2018) suggest that maternal social capital via group memberships is associated with better dietary diversity among children. Whereas, a couple of studies [30,37] find that the effects of health layering on SHGs were relatively weaker on complementary feeding and nutrition. However, much of this evidence is context specific and given the increasing governmental attention on SHGs for women empowerment and livelihoods promotion, it is critical to further the understanding on effectiveness and impact of health layering initiatives on child diets and nutrition.

### The *Parivartan* (Change) initiative

We engage with the health layering experience of the JEEViKA Technical Support Program (JTSP) to draw insights regarding the influence of BCC initiatives on child dietary diversity. To elaborate, JTSP is a partnership program between Bihar Rural Livelihoods Promotion Society (BRLPS), an autonomous body under the Department of Rural Development, Government of Bihar and Project Concern International (PCI), with support from the Bill and Melinda Gates Foundation (BMGF). The initiative is formulated to accelerate health and nutrition improvements in Bihar which has one of the highest prevalence of child undernutrition in India. The NFHS 2019–21 finds that under-five children in Bihar have high prevalence of stunting (43%), underweight (41%) and wasting (23%) and the problem gets intensified because of a high population base of the state. Since 2011, Bihar has experimented with interventions to test the efficacy of health layering interventions for SHGs. In 2012, Project Concern International launched *Parivartan* (translated as ‘change’ in Hindi language) initiative for over 17000 SHGs spread across 55 blocks and 8 districts of Bihar with approvals from the Government of Bihar and funding support from the Bill and Melinda Gates Foundation (BMGF). The broad objective of *Parivartan* was to comprehend the dynamics as well as efficacy in terms of processes and benefits associated with layering of BCC interventions related to reproductive, maternal, new-born, child health, and nutrition (RMNCHN) and sanitation onto the community-based SHG platforms. Usually, SHG activities focus on microfinance and livelihood generation activities but *Parivartan* aimed at engaging with women members of the SHGs as an agent of change at household as well as community level. The strategy was expected to demonstrate discernible influence on behaviours and practices related to health, nutrition and sanitation and thereby render community-wide impact on key indicators. Various evaluations find a positive role of *Parivartan* in influencing health and nutrition outcomes in target communities [30,38]. For instance, Saggurti et al (2018) note significant changes in practices related to family planning, antenatal and newborn care. Mehta et al (2020) also confirms these findings albeit with relatively weaker impact on domains such as complementary feeding practices. Nevertheless, the encouraging results from *Parivartan* motivated PCI to undertake a feasibility test to implement similar interventions among the SHGs formed by JEEViKA (translated as “living or livelihood” in Hindi language)–a local name for the Bihar Rural Livelihoods Project (BRLP) of the Government of Bihar. The feasibility test for health layering was conducted for 9,089 JEEViKA SHGs. Successful efficacy and feasibility test of layering interventions offered a way forward to the conceptualization and launch of the JEEViKA Technical Support Program (JTSP) by the Government of Bihar with support from BMGF. The JTSP operations were rolled out across 101 blocks in 11 districts in 2015–16, 300 blocks in 2016–17 and was scaled up to reach 865,000 SHGs in 534 blocks across all 38 districts by 2019–20.

For JTSP, an important challenge in bringing outcome level change in complementary feeding indicators was the level of rigor in implementing the interventions including identifying and reaching out to target beneficiaries. Based on these considerations, PCI developed a comprehensive package of complementary feeding interventions consisting of BCC Module roll-out that focused on BCC sessions on complementary feeding as well as collective cooking and feeding demonstrations. It is worth mentioning that the BCC module roll out was done by the community mobilizers of JEEViKA group. The JTSP only facilitated the roll out of the BCC module with an objective to ensure exposure of all the SHG (but not necessarily of all the SHG members). Importantly, apart from these, no other additional resources were provided for roll out either by JEEViKA or by JTSP.

The intervention set was captured through a standard operating procedure document, and all facilitators were taken through it with a model process involving role plays and simulations. The tools for BCC to be used at each touch point/ interaction (SHG level flip chart, home visit tool, community event posters and banners, 2nd visit checklist and final ceremony agenda, complete with quizzes, local songs and pledges) were standardized and all facilitators made to practice it before using it at household and community level. ICDS and Health functionaries of the village were encouraged for involvement in home visits and community-based events, with them co-facilitating some of the activities. Reported data informed that they participated in 25% of the activities.

## Data and methods

### BCC module roll out

To test the efficacy of the BCC module (i.e., the complementary feeding SHG session followed by collective cooking and feeding demonstration), a pre-post study design was adopted. The assessment focused on impact of exposure to BCC module roll out on knowledge and practices of complementary feeding behaviour, specifically, dietary diversity among children (6–23 months). These BCC modules help create awareness among SHG members and trigger collective action for the desired behaviour within the group. The BCC intervention consisted of messaging and nudging simple doable actions through 5 interactions or touch points. The first consisted of a SHG level participatory discussion on child feeding practices, among SHG members during their first weekly meeting of the month. As the majority of SHG members present in the meetings were senior women, information was derived from them to list younger pregnant and lactating women in their families. These younger women and their families were visited in their homes, for a second interaction around their current and recommended feeding practices. Community events was then organized by the local village organization (VO)—the federation of 10–12 SHG groups, for a third set of interactions, with the visited young women and their families. In these events, women were encouraged to bring meals prepared for their children, share recipes and experiences around cooking and feeding their children. These events would also have demonstration of food groups (raw foods organized in groups as rangolis), discussion about their benefits, video shows around child feeding practices and collective feeding of young children (aged 6–23 months) by their mothers. The demonstration was conducted at a public place so that all the group members, mothers having child between age of 6–12 months and other opinion makers in the community could participate. Demonstration helped in showcasing the key take-away points of the BCC session and, therefore, acted as positive reinforcement. Following the event, the women were visited again (fourth interaction) in their homes to assess changes in their child feeding practices, particularly to understand whether the previous days’ meals had adequate diversity and frequency. A final and fifth interaction was recognition and rewards ceremony organized by the VO. In this, local ICDS and health functionaries and all young mothers in the village were invited. In this ceremony (PARU—Purak Ahaar Ratna Utsav), women who had reported practicing adequate dietary diversity and frequency were asked to share their experiences and were facilitated for being role models (Purak Ahaar Ratnas).

### Sampling design

The analysis is based on the household survey data collected by PCI in 2017. Two rounds of cross-sectional surveys were undertaken with sampled respondents in pre-intervention phase (baseline reference or comparison group) and post-intervention phase (exposed population or treatment group). The first round (pre-intervention survey) was conducted in September 2017 and was carried out prior to rollout of BCC sessions on complementary feeding. The survey respondents (mothers of children 6–23 months) from this pre-intervention survey are considered as the control group. The post-intervention round i.e., the survey after the roll-out of the BCC module sessions in the target communities was conducted in November 2017. The post-intervention survey has two types of respondents viz. a) the treatment group who were directly exposed to the intervention (i.e., mothers of children 6–23 months who are part of the SHG roll out sessions on BCC modules) and, b) the unexposed or the indirectly exposed group (i.e., other respondents who did not participate in the sessions directly).

A statistical power calculation suggested a desired sample of at least 273 completed interviews with eligible women to detect a minimum 15 percentage point change in the dietary diversity score from an assumed pre-intervention level of 50% point. With an assumption of ~10% non-response, the sample size was revised to be 300 respondents in each round. The sampling adopted a cluster randomization design whereby village organizations (VOs) were considered to be the clusters. A required sample of 60 clusters was estimated for each round on the basis of the general formula for clusters calculation. Since the gap between pre- and post-intervention was about two months, it was pertinent to randomly (without replacement) select 120 VOs (60 VOs for pre- and 60 VOs for post-intervention survey) such that the VOs (or in essence the respondents) selected for the pre-intervention assessment are effectively removed from the sampling frame to avoid possible response bias during the post-intervention survey.

The pre-intervention survey was conducted in the first group of 60 VOs before the BCC sessions were rolled-out. The post-intervention assessment was conducted in the second group of 60 VOs after the roll out of the BCC sessions in those groups. After line-listing of all the 6–23 month old children from the SHG households of each VO, a sample of five children were selected randomly (using random number generator app installed in data collection device) for administering an interview schedule to their mother. The final analytical sample provides diet-related information for 597 children aged 6–23 months (297 children in pre-intervention phase and 300 children in post-intervention phase).

### Outcome variable

Information on 16 food items, that were consumed by child a day before, was categorized into 7 groups as follows: a) grains, roots, tubers and plantains; b) pulses (beans, peas, lentils), nuts and seeds; c) dairy products (milk, infant formula, yogurt, cheese),; d) flesh foods (meat, fish, poultry, organ meats); e) eggs; f) vitamin-A rich fruits and vegetables; and g) other fruits and vegetables (WHO and UNICEF 2021). Information on whether child was being breastfed is also used for creating dietary diversity score. We define dietary diversity as consumption of food from at least 5 out of 8 food groups (including breast milk) following the recent WHO and UNICEF (2021) infant and young child feeding (IYCF) guidelines and is coded as “1” if the child is consuming a diversified diet and “0” otherwise. Nevertheless, as a sensitivity analyses, we also replicate the analysis using the previous classification of adequate dietary diversity defined as consumption of food from at least 4 out of 7 food groups. It is worth noting that the recent definition has expanded the IYCF food groups by considering inclusion of breastfeeding status as an additional food group (WHO and UNICEF 2021).

### Correlates

Our key explanatory variable is direct exposure of the respondent (mother of children 6–23 months) to the BCC module on complementary feeding during the SHGs session roll out. The selection of the socio-economic variables and their categorization is based on commonly defined set of control variables based on a thorough literature review. The key socioeconomic and demographic characteristics of the child are included in the analysis with focus on birth order, maternal age, maternal education, father’s education, social category, household size, occupation of mother, parity, wealth, sanitation, cooking fuel and kitchen garden in household. For analytical purposes, maternal age was coded in three categories viz. a) less than 25 years, b) 25 to 29 years and c) more than 30 years. Maternal and paternal education variable has four categories based on number of years of schooling: no education, 1 to 5 years, 6 to 8 years and more than 9 years. Sex of child is categorized as male and female. Indicator for low birth weight (yes/no) were also included. The social group classification adopted in NFHS surveys are used for categorizing the respondents as: schedule castes (SC), schedule tribes (ST), other backward classes (OBC) and others (comprising of all those who do not belong to SC, ST or OBC categories). Based on the household assets information, a wealth index score was generated using principal component analysis and wealth quintiles were created [39]. Occupation of women has two categories: employed and not employed. Two categories for sanitation variable were created based on whether the household has a toilet. Household size has three categories: less than 5, 5 to 6 and more than 6 members. Parity variable were categorized into 3 groups: 1 to 2 children, 3 to 4 and more than 4 children. Based on average scores across items, the awareness level of mothers in terms of complementary feeding practices was categorised as low and high. Similarly, a score for diet preference for children was categorized as low, medium and high based on the number of food groups preferred to be consumed.

### Data analysis

Cross-tabulations and descriptive statistical analysis are applied to examine the dietary diversity patterns and consumption from different food groups by child by socioeconomic characteristics. The level of dietary diversity is reported with 95% confidence intervals for the food groups and the combined scores. Econometric analysis is used to confirm the association of diversified dietary intake with the socioeconomic characteristics. For this purpose, logistic regression is used that uses the binary variable of adequate dietary diversity (one if adequate and zero otherwise) as the dependent variable, direct exposure to BCC module as the main independent or treatment variable and other socioeconomic variables used for adjusting the associations and confounding.

Finally, for a robust comparison of the impact of the BCC module roll out it is important to compare not only the pre- and post-intervention groups but also to adjust for similarities in terms of their demographic and socioeconomic background. In survey sampling it is plausible that the children in the pre- and post-intervention surveys may not be directly comparable because of the variations in background characteristics of the sample. This can bias the inferences regarding the association of the BCC module exposure with child dietary diversity score. To address such concerns, the propensity score matching (PSM) technique is applied is using the *psmatch2* routine in Stata 15.0 [40–42]. The PSM analysis allows examination of average treatment effect related to the exposed respondents (the treatment group) in the post-intervention phase with same characteristics as respondents from the pre-intervention survey (the comparison group).

#### Ethical clearance

The study was approved by the Ethics Committee of AIIMS Patna. Reference number-10015/IRB/D/17-18. Ethics committee approval for the study was also received from SIGMA Institutional Review Board (IRB) (New Delhi). Verbal consent was taken from the respondents prior to the interview. The IRB had granted the approval to take the verbal consent of the participants during the interviews. All interviews are conducted with the adult participants only and none of the minors are interviewed.

## Results

The study sample comprises of information on dietary diversity of 297 children in the pre-intervention phase and 300 children in the post-intervention phase. The representation of male children in the pre-intervention sample was higher (59%) whereas it was more or less equal in the post-intervention sample (Table 1). To a great extent the background characteristics of the sample respondents and households is homogenous between pre and post intervention. In the pre-intervention sample most of the respondent mothers (61%) and their partners (41%) do not have any formal education or schooling.

**Table 1:** Background characteristics of sample respondents in pre and post-intervention survey, Rural Bihar, 2017.

Background characteristics	Pre-intervention		Post-intervention	
	N	%	N	%
Maternal age				
Less than 25 years	101	34.0	134	44.7
25 to 29 years	124	41.8	113	37.7
More than 30 years	72	24.2	53	17.7
Maternal education				
No education	182	61.3	161	53.7
1 to 5 years	30	10.1	35	11.7
6 to 8 years	34	11.4	32	10.7
More than 9 years	51	17.2	72	24.0
Social group				
SC/ST	87	29.3	89	29.7
OBC and Others	210	70.7	211	70.3
Religion				
Hindu	277	93.3	277	92.3
Muslim and Other	20	6.7	23	7.7
Occupation				
Employed	61	20.5	81	27.0
Not employed	236	79.5	219	73.0
Sex of child (last birth)				
Male	174	58.6	145	48.3
Female	123	41.4	155	51.7
Number of children				
1 to 2	88	29.6	126	42.0
3 to 4	154	51.9	130	43.3
4+	55	18.5	44	14.7
Household with toilet facility				
No	166	55.9	148	49.3
Yes	131	44.1	152	50.7
Cooking fuel				
LPG	90	30.3	74	24.7
Wood	98	33.0	88	29.3
Agricultural crop waste	109	36.7	138	46.0
Kitchen garden				
No	161	54.2	151	50.3
Yes	136	45.8	149	49.7
Child diet knowledge score				
Low (1 to 2)	155	52.2	57	19.0
High (3 to 5)	142	47.8	243	81.0
Child diet preference score				
Low (0 to 2)	44	14.8	16	5.3
Medium (3 to 5)	198	66.7	34	11.3
High (More than 5)	55	18.5	250	83.3
Total	297	100.0	300	100.0

The sample comprises of mostly Hindu households (93% in pre-intervention and 92% in post-intervention sample). Scheduled caste and scheduled tribe (SC/ST) households comprised 30% of the sample in both the survey rounds. Most of the households reported a family size of more than 5 members and the households on average have 3–4 children which indicates high fertility rates. The use of LPG for cooking was low across both the control and intervention areas. Almost one-half of the sample respondents reported knowledge of kitchen garden and reported growing some fruits and vegetables at home or plain land nearby home.

More than two-third (72%) of the sample respondents had attended BCC module session on complementary feeding during the SHG meetings. The remaining 28% are thus unexposed to the BCC intervention in the post-intervention sample. During the pre-intervention phase 48% of the respondents reported higher knowledge levels on complementary feeding practices and this increased to 81% in the post-intervention phase with bulk of this attributable to the exposed group of respondents (91%). Similar pattern in level and changes in diet preference score is also noted whereby low score in dietary preference patterns for children is almost negligible among the exposed group (S2 and S3 Tables).

**Fig 1:**
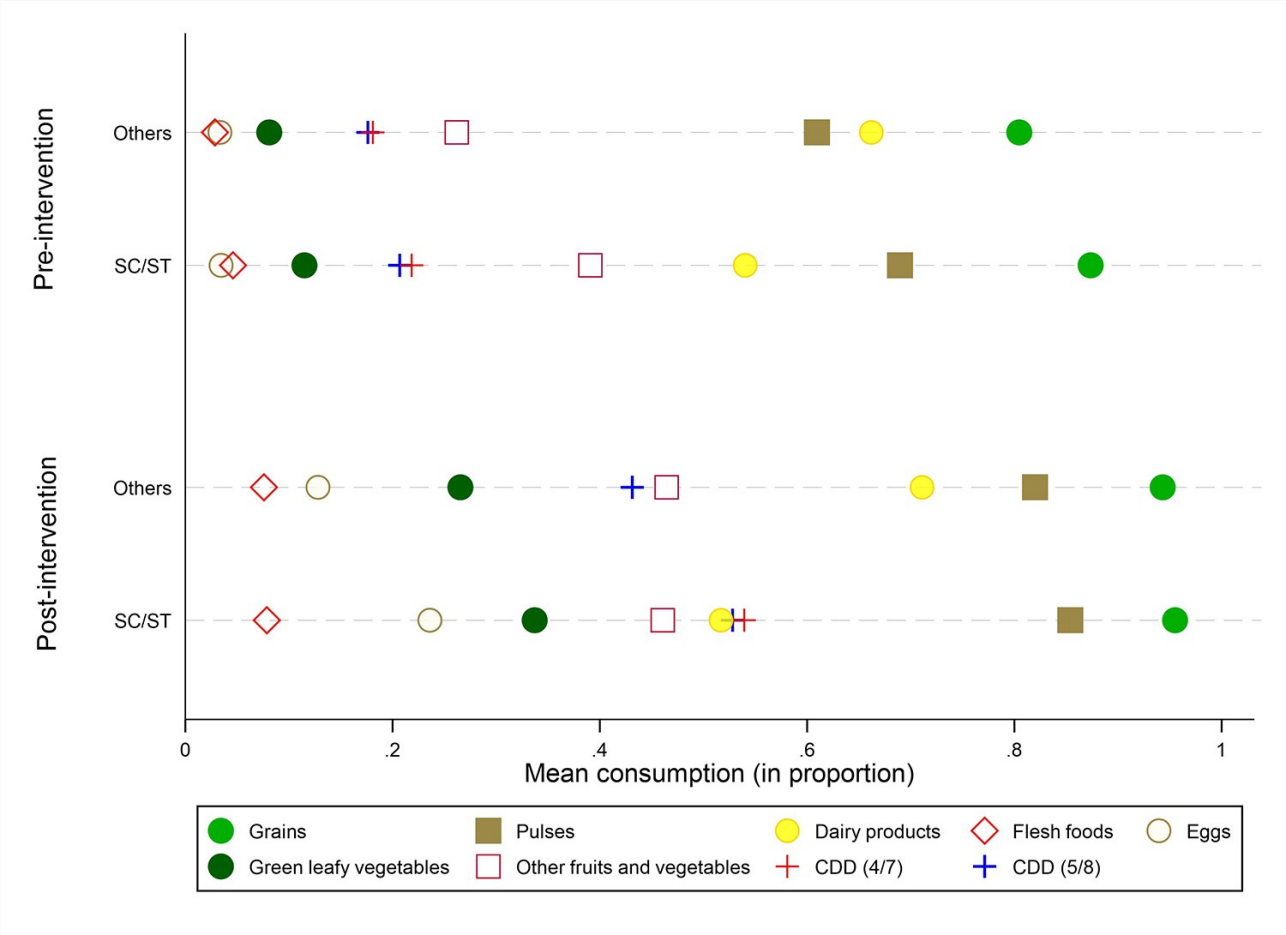
Mean consumption of various food groups by SC-ST and Others during pre- and post-intervention survey, Rural Bihar 2017.

Fig 1 presents the consumption patterns of children 6–23 months from the various food groups (S4 Table). A high proportion of children consume grains, roots, tubers and plantains (83% and 95% in pre- and post-intervention sample, respectively) followed by pulses, beans, nuts and seeds (63% and 83% in pre- and post-intervention sample, respectively). Consumption of eggs among children was 3% in the pre-intervention sample but it increased to 18% among the exposed group in the post-intervention phase (S4 Table). The increase is much higher among SC/ST households than other social groups (Fig 1). Consumption of vitamin A rich fruits and vegetables also improved after the intervention (from 9% to 29%). The child dietary diversity improvements are much higher among the respondents who were exposed to the BCC modules whereas the effect was lower among those who were not directly exposed to these sessions. This indicates that there are likely to be positive but limited spill-over effects of the BCC intervention. Interestingly, no significant changes are noted in the consumption of milk products in the pre- and post-intervention phases. The overall child dietary diversity scores are computed using both the old as well as the new definition of the indicator. As regards the old definition, during the pre-intervention phase, 19% of the children had adequate dietary diversity (eating from at least 4 out of 7 different food groups) and this increased to 49% among the exposed group and to 28% among the non-exposed group in the post-intervention phase. As per the new definition of (consuming from at least 5 out of 8 different food groups including breastfeeding), the estimates of child dietary diversity levels are more or less similar (S4 Table). This is attributable to near universal prevalence of breastfeeding for the 6–23 months old children as revealed through the data from rural Bihar.

Child dietary diversity among children (6–23 months) estimated as consumption of five out of eight food groups is found to be much lower at 19% during the pre-intervention period (Table 2). The levels among respondents exposed (54%) to the BCC module are much better as compared to those who were not exposed (24%) to the module on complementary feeding practices (S4 Table). The dietary diversity levels thus show an over two-fold increase among the exposed group during the post-intervention phase (54%). However, there is only a small increment among the unexposed sample (24%). Both girls (20%) and boys (18%) have similar dietary diversity levels in the pre-intervention sample as well as among the exposed sample in the pre-intervention phase. Nevertheless, in the unexposed sample male child shows a relatively higher level of dietary diversity. Across both control and intervention area, dietary diversity among children is lower in households with more than 4 children. During baseline survey the dietary diversity level among households with and without kitchen garden was more or less similar at 20%.

**Table 2:** Child dietary diversity (5 out of 8 groups) levels in pre and post-intervention survey, Rural Bihar, 2017.

Background characteristics	Dietary Diversity (Pre-intervention)		Dietary Diversity (Post-intervention)	
	N	%	N	%
Maternal age				
Less than 25 years	14	13.9	74	55.2
25 to 29 years	26	21.0	42	37.2
More than 30 years	15	20.8	22	41.5
Maternal education				
No education	28	15.4	60	37.3
1 to 5 years	4	13.3	14	40.0
6 to 8 years	10	29.4	17	53.1
More than 9 years	13	25.5	47	65.3
Social group				
SC/ST	18	20.7	47	52.8
OBC and Others	37	17.6	91	43.1
Religion				
Hindu	51	18.4	129	46.6
Muslim and Other	4	20.0	9	39.1
Occupation				
Employed	12	19.7	38	46.9
Not employed	43	18.2	100	45.7
Sex of child (last birth)				
Male	31	17.8	71	49.0
Female	24	19.5	67	43.2
Number of children				
1 to 2	12	13.6	75	59.5
3 to 4	34	22.1	50	38.5
4+	9	16.4	13	29.5
Household with toilet facility				
No	25	15.1	66	44.6
Yes	30	22.9	72	47.4
Cooking fuel				
LPG	27	30.0	44	59.5
Wood	9	9.2	35	39.8
Agricultural crop waste	19	17.4	59	42.8
Kitchen garden				
No	28	17.4	60	39.7
Yes	27	19.9	78	52.3
Child diet knowledge score				
Low (1 to 2)	21	13.5	15	26.3
High (3 to 5)	34	23.9	123	50.6
Child diet preference score				
Low (0 to 2)	6	13.6	0	0.0
Medium (3 to 5)	32	16.2	10	29.4
High (More than 5)	17	30.9	128	51.2
Total	55	18.5	138	46.0

However, in the post-intervention phase the levels of child dietary diversity is found to be higher among households that have a kitchen garden (57%) than those without kitchen garden (40%). Besides, children belonging to SC/ST category also have higher dietary diversity than those who are other social groups. Dietary diversity is higher among children of mothers in the younger age group of less than 25 years and those who have more than 5 years of education and belong to economically better-off households.

Estimates from the logistic regression analysis confirm a statistically significant association between child dietary diversity and exposure to BCC module during SHG session (S5 and S6 Tables). The odds ratio (OR) in S5 Table shows that exposure to BCC module is associated with 2.90 times (95% confidence interval (CI): 1.46, 5.74) higher chances of child dietary diversity. However, these estimates are adjusted for the complementary feeding knowledge levels as well as household preferences for child diets which itself are influenced because of exposure to the BCC module. This is validated through the increased odds ratio in child dietary diversity among the exposed group when these two variables are removed from the equation (Table 3). The odds ratio increases to 3.81 (95% CI: 2.03, 7.15) when the old definition of 4 out of 7 groups is considered and 4.12 (95% CI: 2.17, 7.81) when new definition of 5 out of 8 groups is considered. The econometric analysis also shows that maternal education has a significant association with child dietary diversity. Children of mothers with 5 or more years of education are twice more likely to consume a diversified diet. Children belonging to SC/ST households have 70% higher chances (OR 1.74, 95% CI: 1.08, 2.79) of consuming a diversified diet. Those who are using LPG are also twice as likely to report a diversified diet (Model-2 OR 2.14, 95% CI: 1.32, 3.46). This indicator presumably captures the wealth effect even though the causation may be difficult as there is a widespread policy effort to ensure universal access to clean cooking fuel implying that some of the poor households may also have LPG cylinders at home through government initiatives such as *Ujjwala*.

**Table 3:** Logistic regression based odds ratio (OR) for child dietary diversity (CDD) for pooled sample, Rural Bihar 2017.

Background characteristics	CDD (4 out of 7 groups)		CDD (5 out of 8 groups)	
	Model-1	Model-2	Model-1	Model-2
Social group: ® OBC / Others	1.00	1.00	1.00	1.00
SC / ST group	1.72*	1.72*	1.85**	1.85**
	[1.09; 2.72]	[1.08; 2.74]	[1.17; 2.93]	[1.16; 2.96]
Maternal education: ® None	1.00	1.00	1.00	1.00
1 to 5 years education	0.88	0.78	0.89	0.79
	[0.44; 1.73]	[0.39; 1.58]	[0.45; 1.76]	[0.39; 1.61]
6 to 8 years education	2.14*	2.21*	2.33*	2.41*
	[1.10; 4.17]	[1.13; 4.34]	[1.20; 4.51]	[1.23; 4.71]
More than 9 years education	2.63**	2.83**	2.41**	2.60**
	[1.39; 4.98]	[1.47; 5.45]	[1.28; 4.55]	[1.36; 5.00]
Sex of child: ® Male	1.00	1.00	1.00	1.00
Female child	0.94	0.96	0.94	0.96
	[0.63; 1.40]	[0.64; 1.44]	[0.63; 1.41]	[0.64; 1.44]
Birth order: ® First or Second	1.00	1.00	1.00	1.00
Third or Fourth	0.73	0.62	0.75	0.64
	[0.43; 1.24]	[0.36; 1.07]	[0.44; 1.28]	[0.37; 1.10]
Four+	0.52	0.45	0.45	0.38*
	[0.22; 1.23]	[0.18; 1.09]	[0.19; 1.07]	[0.16; 0.95]
Wealth quintile: ® Poorest	1.00	1.00	1.00	1.00
Poorer	0.81	0.82	0.88	0.89
	[0.43; 1.53]	[0.43; 1.57]	[0.46; 1.67]	[0.47; 1.72]
Middle	0.92	0.94	0.99	1.02
	[0.48; 1.77]	[0.48; 1.83]	[0.51; 1.91]	[0.52; 1.99]
Richer	0.7	0.7	0.79	0.79
	[0.35; 1.40]	[0.34; 1.41]	[0.39; 1.59]	[0.39; 1.61]
Richest	1.61	1.54	1.74	1.66
	[0.76; 3.43]	[0.71; 3.33]	[0.82; 3.70]	[0.77; 3.60]
Survey: ® Pre-intervention	1.00	1.00	1.00	1.00
Post-intervention	4.59***	1.66	4.27***	1.45
	[3.00; 7.01]	[0.87; 3.17]	[2.79; 6.53]	[0.75; 2.80]
Kitchen garden: ® No	1.00	1.00	1.00	1.00
Yes	1.46	1.35	1.46	1.34
	[0.98; 2.19]	[0.89; 2.03]	[0.98; 2.19]	[0.89; 2.02]
Cooking fuel: ® Non-LPG	1.00	1.00	1.00	1.00
LPG fuel	2.39***	2.34***	2.34***	2.28***
	[1.50; 3.82]	[1.45; 3.76]	[1.47; 3.74]	[1.42; 3.67]
Age of child: ® 6–8 months	1.00	1.00	1.00	1.00
9 to 11 months	1.39	1.53	1.32	1.45
	[0.68; 2.84]	[0.74; 3.17]	[0.64; 2.71]	[0.69; 3.04]
12 to 18 months	2.90***	2.98***	3.07***	3.16***
	[1.57; 5.36]	[1.60; 5.57]	[1.65; 5.69]	[1.68; 5.94]
19 to 23 months	5.58***	5.99***	4.46***	4.77***
	[2.87; 10.87]	[3.03; 11.82]	[2.29; 8.68]	[2.41; 9.44]
Attended BCC in SHG: ® No	-	1.00	-	1.00
Yes	-	3.81***	-	4.12***
		[2.03; 7.15]		[2.17; 7.81]

Note: The models are also adjusted for household size, religion, father’s education, maternal age and maternal occupation. ® denotes the reference group for the odds ratio.

*, ** and *** denotes significance at 10%, 5% and 1% level.

Finally, the impact of the BCC module exposure is assessed using the propensity score matching technique. The ATE and ATT values are estimated for the both old and new definitions of child dietary diversity. The robustness checks for covariate balancing as well as common support for the propensity scores of the matched sample is also performed (S1–S3 Figs and S8 Table). The mean and standard deviation for the propensity score is estimated to 0.422 and 0.111, respectively. The region of common support for the propensity scores ranges from 0.182 to 0.706. For the old definition of child dietary diversity (CDD of 4 out of 7 food groups), the PSM based ATE and ATT estimates of exposure to BCC module during SHG sessions are estimated to be 0.336 [95% CI: 0.246; 0.427] and 0.294 [95% CI: 0.184; 0.404], respectively (Table 4). For the new definition of child dietary diversity (5 out of 8 food groups), the PSM based ATE and ATT estimates are 0.331 [95% CI: 0.240; 0.421] and 0.281 [95% CI: 0.173; 0.389], respectively. The estimates for both CDD definition are also confirmed using the alternative strategy of nearest neighbor matching (Table 4). As sensitivity check for the estimation approach, we present the ATT estimates for both the definitions in S9 Table. The ATT estimates across the various approaches are found to be consistent. The ATT estimate indicates that after matching those children whose mother were exposed to the BCC module have a much higher chance of receiving a diversified diet. If we consider diversity based on 5 out of 8 groups, we observe similar ATT effects.

**Table 4:** Estimated treatment effects of exposure to BCC module for child dietary diversity (CDD) based on propensity score and nearest neighbor matching, Rural Bihar 2017.

Matching technique	CDD (4 out of 7 groups)		CDD (5 out of 8 groups)	
	ATE	ATT	ATE	ATT
Propensity score matching (PSM)	0.336***	0.294***	0.331***	0.281***
	[0.246; 0.427]	[0.184; 0.404]	[0.240; 0.421]	[0.173; 0.389]
Nearest neighbor matching (NNM)	0.340***	0.357***	0.332***	0.343***
	[0.252; 0.429]	[0.261; 0.452]	[0.244; 0.421]	[0.247; 0.439]

Note: The propensity score matching estimates are based on caliper setting of 0.1 i.e. the maximum distance imposed for which two observations are potential neighbors. The nearest neighbor matching is based on one match per observation.

## Discussion and conclusion

The study analyzed the role of BCC module roll out in SHG sessions for improving child dietary diversity in rural Bihar. The five salient findings of the study are as follows: First, compared to the baseline status, the dietary diversity among children 6–23 months showed more than two-fold increase among beneficiaries who were directly exposed to the BCC module roll out at SHG meetings. This effect was robust even after adjusting for socioeconomic factors as well as information on knowledge and dietary preference levels. Further, we find that there was a small increment in dietary diversity scores among the unexposed group but the effect was statistically insignificant. Second, the knowledge about complementary feeding practices and dietary preferences for children are identified as important factors influencing child dietary diversity scores. This effect persisted even after adjusting for the socioeconomic background of the respondents suggesting that behavioral factors can be improved with improving knowledge and awareness on complementary feeding. Third, maternal education level as well as household income status also have independent effects on increasing the chances of providing a more diverse diet to children (6–23 months). This reinforces the association of socioeconomic status with child health and nutrition. Fourth, availability of kitchen gardens, although reveals a favorable effect on child dietary diversity, but the effects were muted when adjusted for various factors in the econometric analysis. It perhaps implies that the pathways to link kitchen gardens with nutritional diversity may have to be viewed on a case-to-case basis during intervention roll out as there is considerable scope of household level variations for nature, type, seasonality and production focus of the kitchen gardens. Finally, the dietary diversity was weaker in terms of provisioning of meat, eggs and green leafy vegetables to children. These preferences can be partly associated with availability concerns and in part because of household preferences and can be verified through further assessments. Provisioning of milk products or fruits was also low. In the post-intervention phase consumption levels of eggs and fruits improved among the exposed group. Similar to other studies, it is noted that demographic factors such as age of child and mother, sex of child and birth order were unrelated with dietary diversity patterns [43]. However, SC/ST households were more likely to have dietary diversity when the recent definition of 5 out of 8 groups was considered. This is found to be associated with improved uptake of eggs as well as higher breastfeeding practices in the SCST community (Table 3).

The study, however, is not devoid of certain limitations. In particular, the study design is based on a pre-post assessment framework with an effort to understand the impact among the exposed and unexposed in the post intervention phase. For this purpose, the sampling of the respondent clusters for both pre-intervention and post-intervention survey was done prior to the launch of the intervention. Although this does not necessarily allow a robust randomized control design but nevertheless a priori random allocation of VOs in two groups makes them comparable with no or limited risk of any self-selection bias. Moreover, the length of intervention being short, about a month, it is least expected that other exogenous factors would affect the outcomes observed between pre- and post-intervention phases. This ‘single’ difference in this design arguably captures the essence of the ‘double’ difference usually adopted in quasi-experimental evaluation designs. Although, the analysis finds that the association of exposure to BCC module on child dietary diversity is significant but given the limitations of the study design it cannot be claimed to be strictly causal association. Furthermore, the post-intervention survey was conducted soon after the BCC module roll out and hence the recall abilities and adopted practices were discerned to be high. It will be important to understand whether such levels are sustained in subsequent months. Also, information on diet quantity is unavailable to understand improvements in actual dietary intake. Finally, it is also important to acknowledge that the BCC effort undertaken by JEEViKA was complementary to the ICDS driven activities like POSHAN *Maah* (month) celebration and monthly community-based events called *Annaprashan Diwas*. However, information on these components were not included in the survey or the analysis. We, however, expect none or minimal influence of the role of ICDS even as it aims at improving dietary diversity with certain basic provisioning of supplementary nutrition services to the beneficiaries (children, pregnant women and lactating mothers). This is mainly because of limited dietary diversity in the supplementary nutrition products distributed by the ICDS.

Overall, the findings suggests that the behavioral change communication in a group setting and through community mobilizers of SHGs can be an important intervention to improve child dietary diversity in rural communities. Along with this, maternal knowledge on complementary feeding practices, maternal education as well as household income are discerned are critical influencing factors in complementary feeding. The theory of change underlying the initiative emphasizes that community group mobilization with focus on community mobilization processes can improve self-efficacy, confidence, collectivization and change in norms and beliefs in accordance with the recommended practices for dietary diversity. In particular, the community mobilization activities were strengthened with both individual level interventions (such as messages and services to the marginalized women) and structural level interventions (such as resources and activities for the community or the group). These in turn were helpful in strengthening program activities such as the coverage of the program and also encouraged participation for trainings, meetings and social advocacy. The increased coverage also led to increase collective agency and action with improvements expected in knowledge levels and behaviors among marginalized women. The collection action also created an enabling environment that supported and reinforced the practices around dietary diversity and nutrition.

The proliferation of community institutions in the form of women collectives or SHGs in rural Bihar and also in other states has paved the way for mobilizing the community and layer health, nutrition, WASH interventions using the SHG cadre such as Community Mobilizers (CMs) and variety of Community Resource Persons (CRPs). While the SHG network is primarily a livelihood platform for rural women, it has complemented the efforts of the frontline health workers e.g., ASHAs, AWWs and ANMs to improve community demand for health, nutrition and WASH services offered by the government. The community institutions have a three tier upwardly federated system. SHGs are at the grassroots level where 10–12 married women form a group and the leaders of 10–12 SHGs form a Village Organization (VO) and the representatives of VOs in a cluster (sub-block unit) form a Cluster Level Federation (CLF). The network is run and managed by the State Rural Livelihood Mission having an office in each block with dedicated staff for overseeing the work of various verticals of the livelihood platform. Interestingly, there are more than 1 million SHG groups in Bihar alone. Nearly 10–12 million rural households have a member in the SHG fold. Hence, any intervention rolled out through the SHG network reaches easily to a large section of the rural community in no time. These SHG members meet once every week. The VO and CLF members meet once a month. Every SHG meeting is usually attended by over 60% of its members. Besides the meeting, the SHG, VO and CLM members and leaders participate in various community level activities and contribute to the local health and development agenda. Thus, it is a promising platform. As part of the organizations, the BCC intervention used multiple community level activities to improve the community engagement besides the module roll out during the weekly SHG meeting (please refer to our description of the intervention for more).

Even in similar other contexts (such as Maharashtra), engagement with BCC modules to improve counseling is found to be effective medium for improving dietary diversity among children from rural and marginalized communities. For instance, a positive change was observed in maternal and child dietary diversity after implementation of ICDS systems strengthening activities including behavior change communication among frontline workers [44]. A few other studies also discern a positive impact on the level of health and nutrition knowledge and feeding practices when BCC module applied through participatory leaning action (PLA) or community groups such as SHGs are introduced [45–49]. Since food and diets are the key determinants of nutritional status, there can be further impacts of such changes in the form of improved anthropometric outcomes among children. For instance, a latest quasi-experimental study in India showed that after the delivery of a knowledge and awareness package to the pregnant mothers, their infants showed significant improvements in mean weight-for-age z-scores [50]. Such evidences support the argument that providing culturally-appropriate nutrition educational intervention to individuals via trained community health workers can effectively improve complementary feeding practices [51].

The analysis also indicated that BCC interventions can augment the knowledge levels on complementary feeding and dietary preferences. However, translation of such knowledge into intent and action i.e., improved dietary diversity can be influenced presumably by socioeconomic status, cultural factors and behavioral norms. This gap between knowledge of complementary feeding and actual practice of child dietary diversity is also observed in this study. This finding is usually common in low-income settings [52]. Sometimes difficulties experienced in child feeding such as refusal by the child despite repeated feeding is a major obstacle for mothers. In fact, studies have noted that forcible feeding sometimes leads to undesirable health consequences such as vomiting which dissuades households to repeat such actions in future [53].

Notwithstanding the role of knowledge, awareness and behaviors, the level of household incomes are perhaps a critical determinant of food availability for women and children [54]. A number of studies from low-income settings confirm the positive association between household incomes and maternal and child dietary diversity [24,37,55,56]. For instance, in West Africa, poverty is seen as a main determinant of cereal-centric diets whereas consumption of meat, fruits and vegetables improve with household socioeconomic status [57]. India, including rural Bihar, is no exception to this basic tenet as noted in this study. A recent study [58] reaffirm that the significant modifiable factors associated with complementary feeding practices includes higher household wealth status and higher maternal education. But heterogeneity of impact across region is also apparent that reinforces the role of locally and culturally-sensitive dietary counseling through community health interventions. Paternal education is a relevant but often neglected aspect in child health and nutrition and should be emphasized as these can lead to improvements in dietary diversity as well as nutrition outcomes among children [36,59]. This also provides programmatic insights for BCC centric interventions for inclusion of men (husbands and fathers) in such SHG session roll outs through alternate participatory mechanisms.

There are many studies that linked the effectiveness of kitchen garden in enhancing dietary diversity [60]. For instance, kitchen gardens were observed to significantly influence dietary diversity among landless households in Myanmar and enhances food security of households [61]. There is robust evidence from other countries such as Kenya and Burkina Faso that on community participatory approach toward agriculture and nutrition activities have influenced dietary diversity but the impact of kitchen gardens per se have been small or minimal [62,63]. Our analysis in the context of rural Bihar also shows that the success of kitchen gardens in influencing dietary diversity is not necessarily universal and it can be rather context-driven. Such findings are consistent with other studies where lack of significant association between home-gardens and dietary diversity are observed. A potential caveat in such interpretations, however, is the nature of study design and the sample size requirements for estimation of the effects [64]. For instance, dietary diversity levels among children is notably higher during rainy season but such studies are exception as often cropping pattern as well as seasonality are not considered while drawing such conclusions [65].

Our study finds that demographic characteristics of the children do not majorly impact dietary diversity. Low or no disparities across demographic features is a common understanding in the literature where age and sex of the child are often unrelated with dietary diversity score [64]. However, within the age group of 6–23 months, some age-related disadvantages in dietary diversity is apparent for the younger 6–8 months old infants [66]. Gender disparity in dietary intake is also found to be minimal or negligible. In fact, in the South Asian as well as Indian context there is low intra-household variation in food consumption among the marginalized communities [67,68].

Finally, in the Indian context, it is observed that there is a low preference for feeding meat, eggs and green leafy vegetables to children [69]. Provisioning of milk products or fruits was also low. These preferences can be partly associated with availability concerns and in part because of household preferences even as only less than one-third of the Indian population are found to strictly vegetarian [70,71]. However, some of these practices for children can be improved as noted in the post-intervention phase findings of this study where an increase in the consumption of eggs and fruits among the exposed group is apparent. Our findings suggest SC/ST households have small relative advantage in dietary diversity which can be attributable to varied food preferences as well as access to various non-market sources of food such as forests and backyard poultry [72]. Nevertheless, encouraging non-vegetarian diet for children can be a formidable task for behavior change strategies both because of availability concern as well as preference considerations. Recent research has also highlighted the question of affordability of such diets for the low-income households. For instance, Gupta et al (2021) estimate that it would cost $1.00 per person per day more on each of the food groups meat, fish, poultry, dairy foods and fruits to meet the recommendations on dietary intake as put forward by the EAT Lancet Commission. Further work on strengthening dietary diversity patterns and improving availability and affordability is necessary as these often persist as a lifelong practice and can hamper nutrition in later life [73].

In conclusion, it may be reiterated that there is substantial scope to improve child dietary diversity across rural communities in India with the help of BCC modules rolled out in community settings such as the SHGs. The improvements noted in the levels of dietary diversity in a relatively shorter time span suggests that such patterns are often shaped by behavioral practices that can be altered through effective counseling of mothers and community outreach for wider understanding of nutrition concerns. The reductions in childhood stunting and underweight in Bihar between NFHS 2015–16 and 2019–21 is perhaps a testimony that widespread community-based engagements can help initiate the cycle of improvements almost instantaneously. The SHGs also appear to be a pertinent vehicle as there is a greater governmental involvement and support for health and nutrition layering through women empowerment initiatives (such as National Rural Livelihood Mission, NRLM) across several states of India. Such platform simultaneously can also overcome resource constraints through leveraging economic activities for improving food security and incomes at the household level. However, further research attention is warranted on issues related to sustainability of outcomes and impact demonstrated by such initiatives in rural areas. Also, strategies underlying the BCC roll out would need further research attention to inform policymaking and interventions to reduce heterogeneity of impacts observed across contexts.

## Supporting information

S1 Fig Propensity score overlap plot for treated and comparison group. (JPG)

S2 Fig Box plot for propensity score distribution of treated and comparison groups. (JPG)

S3 Fig Balance plot for propensity score density of treated and comparison groups. (JPG)

S1 Table Socio-economic distribution, intervention and control areas, Household Survey, Bihar. (DOCX)

S2 Table Intake of diversified diet (5 out of 8 groups) by socio-economic distribution, intervention and control areas, Household Survey, Bihar. (DOCX)

S3 Table Intake of diversified diet (4 out of 7 groups) by socio-economic distribution, intervention and control areas, Household Survey, Bihar. (DOCX)

S4 Table Intake of various food items, intervention and control area, Household Survey, Bihar. (DOCX)

S5 Table Logistic regression results, child dietary diversity (5 out of 8 groups). (DOCX)

S6 Table Logistic regression results, child dietary diversity (4 out of 7 groups). (DOCX)

S7 Table Logistic regression results, child dietary diversity excluding knowledge and preference score. (DOCX)

S8 Table Sensitivity analysis based on alternative estimators for the ATT effects for child dietary diversity. (DOCX)

S9 Table Sensitivity analysis based on alternative estimators for the ATT effects for child dietary diversity. (DOCX)

S1 Text (DOCX)

S1 Data (ZIP)
