# Supplementary figures and images for: Association of BCC Module Roll-Out in SHG meetings with changes in complementary feeding and dietary diversity among children (6–23 months)? Evidence from JEEViKA in Rural Bihar, India
Source: PLoS One. 2023 Jan 5;18(1):e0279724. doi: 10.1371/journal.pone.0279724 (PMC9815627; doi:10.1371/journal.pone.0279724)

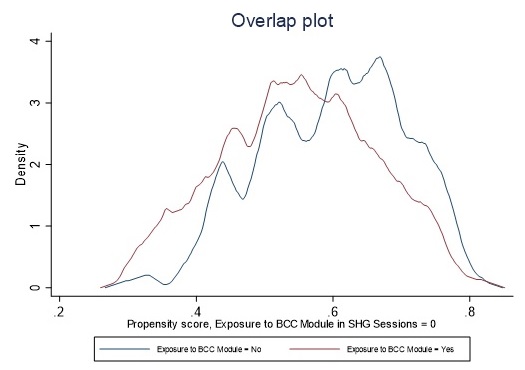

Supplement: S1 Fig — (JPG) [file pone.0279724.s001.jpg]

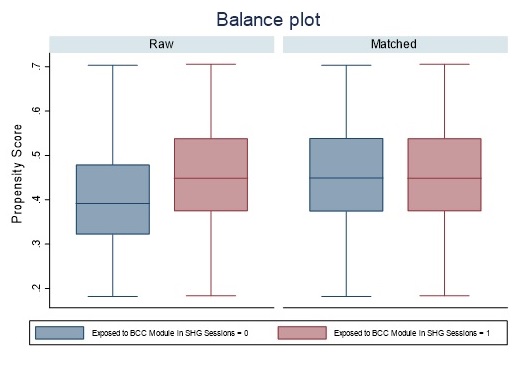

Supplement: S2 Fig — (JPG) [file pone.0279724.s002.jpg]

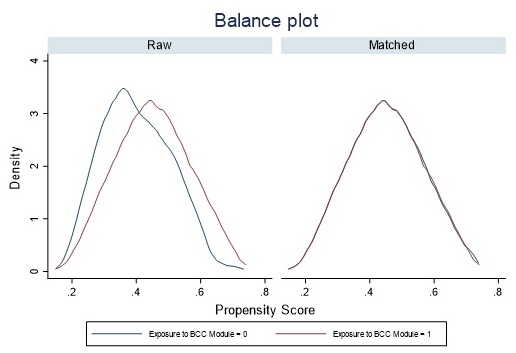

Supplement: S3 Fig — (JPG) [file pone.0279724.s003.jpg]
