# Supplementary material for: Association of BCC Module Roll-Out in SHG meetings with changes in complementary feeding and dietary diversity among children (6–23 months)? Evidence from JEEViKA in Rural Bihar, India
Source: PLoS One. 2023 Jan 5;18(1):e0279724. doi: 10.1371/journal.pone.0279724 (PMC9815627; doi:10.1371/journal.pone.0279724)
Supplement: S1 Table — (DOCX) [file pone.0279724.s004.docx]

**Supplementary Table S1:** Socio-economic distribution, intervention and control areas, Household Survey, Bihar

| **Background characteristics** | **Control** | | **Intervention** | | **Exposed** | | **Not Exposed** | |
| --- | --- | --- | --- | --- | --- | --- | --- | --- |
|  | **N** | **%** | **No.** | **%** | **No.** | **%** | **No.** | **%** |
| Household Size |  |  |  |  |  |  |  |  |
| Less than 5 | 58 | 19.5 | 73 | 24.3 | 57 | 26.3 | 16 | 19.3 |
| 5 to 6 | 125 | 42.1 | 121 | 40.3 | 82 | 37.8 | 39 | 47 |
| Greater than 6 | 114 | 38.4 | 106 | 35.3 | 78 | 35.9 | 28 | 33.7 |
| Religion |  |  |  |  |  |  |  |  |
| Hindu | 277 | 93.3 | 277 | 92.3 | 204 | 94 | 73 | 88 |
| Muslim and Other | 20 | 6.7 | 23 | 7.7 | 13 | 6 | 10 | 12 |
| Social group |  |  |  |  |  |  |  |  |
| OBC and Other | 210 | 70.7 | 211 | 70.3 | 150 | 69.1 | 61 | 73.5 |
| SC/ST | 87 | 29.3 | 89 | 29.7 | 67 | 30.9 | 22 | 26.5 |
| Education |  |  |  |  |  |  |  |  |
| No education | 182 | 61.3 | 161 | 53.7 | 115 | 53 | 46 | 55.4 |
| 1 to 5 years | 30 | 10.1 | 35 | 11.7 | 28 | 12.9 | 7 | 8.4 |
| 6 to 8 years | 34 | 11.4 | 32 | 10.7 | 24 | 11.1 | 8 | 9.6 |
| More than 9 years | 51 | 17.2 | 72 | 24 | 50 | 23 | 22 | 26.5 |
| Husband's education |  |  |  |  |  |  |  |  |
| No education | 121 | 40.7 | 126 | 42 | 89 | 41 | 37 | 44.6 |
| 1 to 5 years | 36 | 12.1 | 40 | 13.3 | 27 | 12.4 | 13 | 15.7 |
| 6 to 8 years | 57 | 19.2 | 46 | 15.3 | 36 | 16.6 | 10 | 12 |
| More than 9 years | 83 | 27.9 | 88 | 29.3 | 65 | 30 | 23 | 27.7 |
| Age |  |  |  |  |  |  |  |  |
| Less than 25 years | 101 | 34 | 134 | 44.7 | 95 | 43.8 | 39 | 47 |
| 25 to 29 years | 124 | 41.8 | 113 | 37.7 | 80 | 36.9 | 33 | 39.8 |
| More than 30 years | 72 | 24.2 | 53 | 17.7 | 42 | 19.4 | 11 | 13.3 |
| Occupation |  |  |  |  |  |  |  |  |
| Employed | 61 | 20.5 | 81 | 27 | 60 | 27.6 | 21 | 25.3 |
| Not employed | 236 | 79.5 | 219 | 73 | 157 | 72.4 | 62 | 74.7 |
| Sex of child |  |  |  |  |  |  |  |  |
| Male | 174 | 58.6 | 145 | 48.3 | 108 | 49.8 | 37 | 44.6 |
| Female | 123 | 41.4 | 155 | 51.7 | 109 | 50.2 | 46 | 55.4 |
| Number of children |  |  |  |  |  |  |  |  |
| 1 to 2 | 88 | 29.6 | 126 | 42 | 84 | 38.7 | 42 | 50.6 |
| 3 to 4 | 154 | 51.9 | 130 | 43.3 | 101 | 46.5 | 29 | 34.9 |
| 4+ | 55 | 18.5 | 44 | 14.7 | 32 | 14.7 | 12 | 14.5 |
| Age of child |  |  |  |  |  |  |  |  |
| 6 to 8 months | 65 | 21.9 | 45 | 15.0 | 34 | 15.7 | 11 | 13.3 |
| 9 to 11 months | 58 | 19.5 | 56 | 18.7 | 36 | 16.6 | 20 | 24.1 |
| 12 to 18 months | 120 | 40.4 | 122 | 40.7 | 90 | 41.5 | 32 | 38.6 |
| More than 18 months | 54 | 18.2 | 77 | 25.7 | 57 | 26.3 | 20 | 24.1 |
| Household have toilet facility |  |  |  |  |  |  |  |  |
| No | 166 | 55.9 | 148 | 49.3 | 110 | 50.7 | 38 | 45.8 |
| Yes | 131 | 44.1 | 152 | 50.7 | 107 | 49.3 | 45 | 54.2 |
| Cooking fuel in your household |  |  |  |  |  |  |  |  |
| LPG | 90 | 30.3 | 74 | 24.7 | 58 | 26.7 | 16 | 19.3 |
| Wood | 98 | 33 | 88 | 29.3 | 63 | 29 | 25 | 30.1 |
| Agricultural crop waste | 109 | 36.7 | 138 | 46 | 96 | 44.2 | 42 | 50.6 |
| Wealth |  |  |  |  |  |  |  |  |
| poorest | 60 | 20.2 | 60 | 20 | 43 | 19.8 | 17 | 20.5 |
| poorer | 59 | 19.9 | 60 | 20 | 43 | 19.8 | 17 | 20.5 |
| middle | 61 | 20.5 | 61 | 20.3 | 42 | 19.4 | 19 | 22.9 |
| richer | 58 | 19.5 | 59 | 19.7 | 42 | 19.4 | 17 | 20.5 |
| richest | 59 | 19.9 | 60 | 20 | 47 | 21.7 | 13 | 15.7 |
| Kitchen garden |  |  |  |  |  |  |  |  |
| No | 161 | 54.2 | 151 | 50.3 | 101 | 46.5 | 50 | 60.2 |
| Yes | 136 | 45.8 | 149 | 49.7 | 116 | 53.5 | 33 | 39.8 |
| Knowledge score |  |  |  |  |  |  |  |  |
| Low (1 to 2) | 155 | 52.2 | 57 | 19 | 20 | 9.2 | 37 | 44.6 |
| High (3 to 5) | 142 | 47.8 | 243 | 81 | 197 | 90.8 | 46 | 55.4 |
| Child diet preference score |  |  |  |  |  |  |  |  |
| Low (0 to 2) | 44 | 14.8 | 16 | 5.3 | 1 | 0.5 | 15 | 18.1 |
| Medium (3 to 5) | 198 | 66.7 | 34 | 11.3 | 12 | 5.5 | 22 | 26.5 |
| High (More than 5) | 55 | 18.5 | 250 | 83.3 | 204 | 94 | 46 | 55.4 |
| Attended CF (session / module) |  |  |  |  |  |  |  |  |
| Yes | - | - | 217 | 72.3 | - | - | - | - |
| No | - | - | 83 | 27.7 | - | - | - | - |
| Total | 297 | 100 | 300 | 100 | 217 | 100 | 83 | 100 |
