# Supplementary material for: Association of BCC Module Roll-Out in SHG meetings with changes in complementary feeding and dietary diversity among children (6–23 months)? Evidence from JEEViKA in Rural Bihar, India
Source: PLoS One. 2023 Jan 5;18(1):e0279724. doi: 10.1371/journal.pone.0279724 (PMC9815627; doi:10.1371/journal.pone.0279724)
Supplement: S2 Table — (DOCX) [file pone.0279724.s005.docx]

**Supplementary Table S2:** Intake of diversified diet (5 out of 8 groups) by socio-economic distribution, intervention and control areas, Household Survey, Bihar

| **Background characteristics** | **Control** | | **Intervention** | | **Exposed** | | **Not Exposed** | |
| --- | --- | --- | --- | --- | --- | --- | --- | --- |
|  | **No.** | **%** | **No.** | **%** | **No.** | **%** | **No.** | **%** |
| Household Size |  |  |  |  |  |  |  |  |
| Less than 5 | 8 | 13.8 | 39 | 53.4 | 35 | 61.4 | 4 | 25 |
| 5 to 6 | 23 | 18.4 | 55 | 45.5 | 43 | 52.4 | 12 | 30.8 |
| Greater than 6 | 24 | 21.1 | 44 | 41.5 | 40 | 51.3 | 4 | 14.3 |
| Religion |  |  |  |  |  |  |  |  |
| Hindu | 51 | 18.4 | 129 | 46.6 | 110 | 53.9 | 19 | 26 |
| Muslim and Other | 4 | 20 | 9 | 39.1 | 8 | 61.5 | 1 | 10 |
| Social group |  |  |  |  |  |  |  |  |
| OBC and Other | 37 | 17.6 | 91 | 43.1 | 79 | 52.7 | 12 | 19.7 |
| SC/ST | 18 | 20.7 | 47 | 52.8 | 39 | 58.2 | 8 | 36.4 |
| Education |  |  |  |  |  |  |  |  |
| No education | 28 | 15.4 | 60 | 37.3 | 53 | 46.1 | 7 | 15.2 |
| 1 to 5 years | 4 | 13.3 | 14 | 40 | 14 | 50 | 0 | 0 |
| 6 to 8 years | 10 | 29.4 | 17 | 53.1 | 13 | 54.2 | 4 | 50 |
| More than 9 years | 13 | 25.5 | 47 | 65.3 | 38 | 76 | 9 | 40.9 |
| Husband's education |  |  |  |  |  |  |  |  |
| No education | 20 | 16.5 | 45 | 35.7 | 39 | 43.8 | 6 | 16.2 |
| 1 to 5 years | 6 | 16.7 | 17 | 42.5 | 15 | 55.6 | 2 | 15.4 |
| 6 to 8 years | 10 | 17.5 | 29 | 63 | 27 | 75 | 2 | 20 |
| More than 9 years | 19 | 22.9 | 47 | 53.4 | 37 | 56.9 | 10 | 43.5 |
| Age |  |  |  |  |  |  |  |  |
| Less than 25 years | 14 | 13.9 | 74 | 55.2 | 62 | 65.3 | 12 | 30.8 |
| 25 to 29 years | 26 | 21 | 42 | 37.2 | 37 | 46.3 | 5 | 15.2 |
| More than 30 years | 15 | 20.8 | 22 | 41.5 | 19 | 45.2 | 3 | 27.3 |
| Occupation |  |  |  |  |  |  |  |  |
| Employed | 12 | 19.7 | 38 | 46.9 | 33 | 55 | 5 | 23.8 |
| Not employed | 43 | 18.2 | 100 | 45.7 | 85 | 54.1 | 15 | 24.2 |
| Sex of child |  |  |  |  |  |  |  |  |
| Male | 31 | 17.8 | 71 | 49 | 58 | 53.7 | 13 | 35.1 |
| Female | 24 | 19.5 | 67 | 43.2 | 60 | 55 | 7 | 15.2 |
| Number of children |  |  |  |  |  |  |  |  |
| 1 to 2 | 12 | 13.6 | 75 | 59.5 | 62 | 73.8 | 13 | 31 |
| 3 to 4 | 34 | 22.1 | 50 | 38.5 | 45 | 44.6 | 5 | 17.2 |
| 4+ | 9 | 16.4 | 13 | 29.5 | 11 | 34.4 | 2 | 16.7 |
| Age of child |  |  |  |  |  |  |  |  |
| 6 to 8 months | 7 | 10.8 | 14 | 31.1 | 12 | 35.3 | 2 | 18.2 |
| 9 to 11 months | 4 | 6.9 | 22 | 39.3 | 20 | 55.6 | 2 | 10 |
| 12 to 18 months | 28 | 23.3 | 57 | 46.7 | 47 | 52.2 | 10 | 31.3 |
| More than 18 months | 16 | 29.6 | 45 | 58.4 | 39 | 68.4 | 6 | 30 |
| Household have toilet facility |  |  |  |  |  |  |  |  |
| No | 25 | 15.1 | 66 | 44.6 | 55 | 50 | 11 | 28.9 |
| Yes | 30 | 22.9 | 72 | 47.4 | 63 | 58.9 | 9 | 20 |
| Cooking fuel in your household |  |  |  |  |  |  |  |  |
| LPG | 27 | 30 | 44 | 59.5 | 38 | 65.5 | 6 | 37.5 |
| Wood | 9 | 9.2 | 35 | 39.8 | 29 | 46 | 6 | 24 |
| Agricultural crop waste | 19 | 17.4 | 59 | 42.8 | 51 | 53.1 | 8 | 19 |
| Wealth |  |  |  |  |  |  |  |  |
| poorest | 8 | 13.3 | 24 | 40 | 22 | 51.2 | 2 | 11.8 |
| poorer | 10 | 16.9 | 24 | 40 | 19 | 44.2 | 5 | 29.4 |
| middle | 9 | 14.8 | 26 | 42.6 | 22 | 52.4 | 4 | 21.1 |
| richer | 6 | 10.3 | 27 | 45.8 | 22 | 52.4 | 5 | 29.4 |
| richest | 22 | 37.3 | 37 | 61.7 | 33 | 70.2 | 4 | 30.8 |
| Kitchen garden |  |  |  |  |  |  |  |  |
| No | 28 | 17.4 | 60 | 39.7 | 52 | 51.5 | 8 | 16 |
| Yes | 27 | 19.9 | 78 | 52.3 | 66 | 56.9 | 12 | 36.4 |
| Knowledge score |  |  |  |  |  |  |  |  |
| Low (1 to 2) | 21 | 13.5 | 15 | 26.3 | 8 | 40 | 7 | 18.9 |
| High (3 to 5) | 34 | 23.9 | 123 | 50.6 | 110 | 55.8 | 13 | 28.3 |
| Child diet preference score |  |  |  |  |  |  |  |  |
| Low (0 to 2) | 6 | 13.6 | 0 | 0 | 0 | 0 | 0 | 0 |
| Medium (3 to 5) | 32 | 16.2 | 10 | 29.4 | 6 | 50 | 4 | 18.2 |
| High (More than 5) | 17 | 30.9 | 128 | 51.2 | 112 | 54.9 | 16 | 34.8 |
| Attended CF (session / module) |  |  |  |  |  |  |  |  |
| yes | - | - | 118 | 54.4 | - | - | - | - |
| no | - | - | 20 | 24.1 | - | - | - | - |
| Total | 55 | 18.5 | 138 | 46 | 118 | 54.4 | 20 | 24.1 |
