# Supplementary material for: Association of BCC Module Roll-Out in SHG meetings with changes in complementary feeding and dietary diversity among children (6–23 months)? Evidence from JEEViKA in Rural Bihar, India
Source: PLoS One. 2023 Jan 5;18(1):e0279724. doi: 10.1371/journal.pone.0279724 (PMC9815627; doi:10.1371/journal.pone.0279724)
Supplement: S3 Table — (DOCX) [file pone.0279724.s006.docx]

**Supplementary Table S3:** Intake of diversified diet (4 out of 7 groups) by socio-economic distribution, intervention and control areas, Household Survey, Bihar

| **Background characteristics** | Control | | Intervention | | Exposed | | Not Exposed | |
| --- | --- | --- | --- | --- | --- | --- | --- | --- |
|  | N | % | N | % | N | % | N | % |
| Household Size |  |  |  |  |  |  |  |  |
| Less than 5 | 8 | 13.8 | 42 | 57.5 | 37 | 64.9 | 5 | 31.3 |
| 5 to 6 | 24 | 19.2 | 59 | 48.8 | 45 | 54.9 | 14 | 35.9 |
| Greater than 6 | 25 | 21.9 | 45 | 42.5 | 41 | 52.6 | 4 | 14.3 |
| Religion |  |  |  |  |  |  |  |  |
| Hindu | 53 | 19.1 | 137 | 49.5 | 115 | 56.4 | 22 | 30.1 |
| Muslim and Other | 4 | 20 | 9 | 39.1 | 8 | 61.5 | 1 | 10 |
| Social group |  |  |  |  |  |  |  |  |
| OBC and Other | 38 | 18.1 | 98 | 46.4 | 83 | 55.3 | 15 | 24.6 |
| SC/ST | 19 | 21.8 | 48 | 53.9 | 40 | 59.7 | 8 | 36.4 |
| Education |  |  |  |  |  |  |  |  |
| No education | 29 | 15.9 | 64 | 39.8 | 57 | 49.6 | 7 | 15.2 |
| 1 to 5 years | 4 | 13.3 | 15 | 42.9 | 15 | 53.6 | 0 | 0 |
| 6 to 8 years | 10 | 29.4 | 17 | 53.1 | 13 | 54.2 | 4 | 50 |
| More than 9 years | 14 | 27.5 | 50 | 69.4 | 38 | 76 | 12 | 54.5 |
| Husband's education |  |  |  |  |  |  |  |  |
| No education | 21 | 17.4 | 49 | 38.9 | 43 | 48.3 | 6 | 16.2 |
| 1 to 5 years | 6 | 16.7 | 17 | 42.5 | 15 | 55.6 | 2 | 15.4 |
| 6 to 8 years | 11 | 19.3 | 30 | 65.2 | 27 | 75 | 3 | 30 |
| More than 9 years | 19 | 22.9 | 50 | 56.8 | 38 | 58.5 | 12 | 52.2 |
| Age |  |  |  |  |  |  |  |  |
| Less than 25 years | 15 | 14.9 | 78 | 58.2 | 64 | 67.4 | 14 | 35.9 |
| 25 to 29 years | 27 | 21.8 | 45 | 39.8 | 39 | 48.8 | 6 | 18.2 |
| More than 30 years | 15 | 20.8 | 23 | 43.4 | 20 | 47.6 | 3 | 27.3 |
| Occupation |  |  |  |  |  |  |  |  |
| Employed | 13 | 21.3 | 39 | 48.1 | 34 | 56.7 | 5 | 23.8 |
| Not employed | 44 | 18.6 | 107 | 48.9 | 89 | 56.7 | 18 | 29 |
| Sex of child |  |  |  |  |  |  |  |  |
| Male | 32 | 18.4 | 75 | 51.7 | 61 | 56.5 | 14 | 37.8 |
| Female | 25 | 20.3 | 71 | 45.8 | 62 | 56.9 | 9 | 19.6 |
| Number of children |  |  |  |  |  |  |  |  |
| 1 to 2 | 13 | 14.8 | 79 | 62.7 | 63 | 75 | 16 | 38.1 |
| 3 to 4 | 34 | 22.1 | 53 | 40.8 | 48 | 47.5 | 5 | 17.2 |
| 4+ | 10 | 18.2 | 14 | 31.8 | 12 | 37.5 | 2 | 16.7 |
| Age of child |  |  |  |  |  |  |  |  |
| 6 to 8 months | 8 | 12.3 | 14 | 31.1 | 12 | 35.3 | 2 | 18.2 |
| 9 to 11 months | 4 | 6.9 | 24 | 42.9 | 21 | 58.3 | 3 | 15 |
| 12 to 18 months | 28 | 23.3 | 57 | 46.7 | 47 | 52.2 | 10 | 31.3 |
| More than 18 months | 17 | 31.5 | 51 | 66.2 | 43 | 75.4 | 8 | 40 |
| Household have toilet facility |  |  |  |  |  |  |  |  |
| No | 27 | 16.3 | 68 | 45.9 | 57 | 51.8 | 11 | 28.9 |
| Yes | 30 | 22.9 | 78 | 51.3 | 66 | 61.7 | 12 | 26.7 |
| Exclusive use of mobile phone |  |  |  |  |  |  |  |  |
| No | 12 | 12.6 | 41 | 47.7 | 33 | 60 | 8 | 25.8 |
| Yes | 45 | 22.3 | 105 | 49.1 | 90 | 55.6 | 15 | 28.8 |
| Cooking fuel in your household |  |  |  |  |  |  |  |  |
| LPG | 28 | 31.1 | 46 | 62.2 | 39 | 67.2 | 7 | 43.8 |
| Wood | 9 | 9.2 | 38 | 43.2 | 32 | 50.8 | 6 | 24 |
| Agricultural crop waste | 20 | 18.3 | 62 | 44.9 | 52 | 54.2 | 10 | 23.8 |
| Wealth |  |  |  |  |  |  |  |  |
| poorest | 8 | 13.3 | 27 | 45 | 25 | 58.1 | 2 | 11.8 |
| poorer | 10 | 16.9 | 26 | 43.3 | 20 | 46.5 | 6 | 35.3 |
| middle | 10 | 16.4 | 27 | 44.3 | 23 | 54.8 | 4 | 21.1 |
| richer | 7 | 12.1 | 27 | 45.8 | 22 | 52.4 | 5 | 29.4 |
| richest | 22 | 37.3 | 39 | 65 | 33 | 70.2 | 6 | 46.2 |
| Kitchen garden |  |  |  |  |  |  |  |  |
| No | 29 | 18 | 64 | 42.4 | 55 | 54.5 | 9 | 18 |
| Yes | 28 | 20.6 | 82 | 55 | 68 | 58.6 | 14 | 42.4 |
| Knowledge score |  |  |  |  |  |  |  |  |
| Low (1 to 2) | 23 | 14.8 | 16 | 28.1 | 8 | 40 | 8 | 21.6 |
| High (3 to 5) | 34 | 23.9 | 130 | 53.5 | 115 | 58.4 | 15 | 32.6 |
| Child diet preference score |  |  |  |  |  |  |  |  |
| Low (0 to 2) | 7 | 15.9 | 1 | 6.3 | 0 | 0 | 1 | 6.7 |
| Medium (3 to 5) | 33 | 16.7 | 11 | 32.4 | 7 | 58.3 | 4 | 18.2 |
| High (More than 5) | 17 | 30.9 | 134 | 53.6 | 116 | 56.9 | 18 | 39.1 |
| Attended complementary feeding (session / module) |  |  |  |  |  |  |  |  |
| yes | - | - | 123 | 56.7 | - | - | - | - |
| no | - | - | 23 | 27.7 | - | - | - | - |
| Total | 57 | 19.2 | 146 | 48.7 | 123 | 56.7 | 23 | 27.7 |
