# Supplementary material for: Association of BCC Module Roll-Out in SHG meetings with changes in complementary feeding and dietary diversity among children (6–23 months)? Evidence from JEEViKA in Rural Bihar, India
Source: PLoS One. 2023 Jan 5;18(1):e0279724. doi: 10.1371/journal.pone.0279724 (PMC9815627; doi:10.1371/journal.pone.0279724)
Supplement: S4 Table — (DOCX) [file pone.0279724.s007.docx]

**Supplementary Table S4:** Intake of various food items, intervention and control area, Household Survey, Bihar

| **Food items** | **Control** | |  | **Intervention** | |  | **Exposed** | |  | **Not Exposed** | |
| --- | --- | --- | --- | --- | --- | --- | --- | --- | --- | --- | --- |
|  | **N** | **%** | **N** | **%** | **N** | **%** | **N** | **%** |  | **N** | **%** |
| Overall |  |  |  |  |  |  |  |  |  |  |  |
| Grains | 82.5 | [77.7;86.4] |  | 94.7 | [91.5;96.7] |  | 95.4 | [91.6;97.5] |  | 92.8 | [84.5;96.8] |
| Pulses | 63.3 | [57.6;68.6] |  | 83.0 | [78.3;86.8] |  | 87.1 | [81.9;91] |  | 72.3 | [61.5;81] |
| Dairy products | 62.6 | [57;68] |  | 65.3 | [59.8;70.5] |  | 69.1 | [62.6;75] |  | 55.4 | [45;65.4] |
| Flesh foods | 3.4 | [1.8;6.2] |  | 7.7 | [5.2;11.2] |  | 9.7 | [6.4;14.4] |  | 2.4 | [0.6;9.5] |
| Egg | 3.4 | [1.8;6.2] |  | 16.0 | [12.3;20.6] |  | 18.0 | [13.4;23.6] |  | 10.8 | [5.7;19.6] |
| Green leafy vegetables | 9.1 | [6.3;12.9] |  | 28.7 | [23.8;34.1] |  | 34.6 | [28.5;41.2] |  | 13.3 | [7.4;22.7] |
| Other fruits and vegetables | 30.0 | [25.1;35.4] |  | 46.3 | [40.8;52] |  | 51.6 | [44.9;58.2] |  | 32.5 | [23.6;42.9] |
| Breastfeeding | 97.3 | [94.7;98.7] |  | 92.7 | [89.1;95.1] |  | 94.9 | [91;97.2] |  | 86.8 | [77.4;92.6] |
| CDD (4/7 groups) | 19.2 | [15.1;24.1] |  | 48.7 | [43.1;54.3] |  | 56.7 | [50;63.2] |  | 27.7 | [19.2;38.2] |
| CDD (5/8 groups) | 18.5 | [14.5;23.4] |  | 46.0 | [40.5;51.6] |  | 54.4 | [47.7;60.9] |  | 24.1 | [16.3;34.1] |
| SC/ST |  |  |  |  |  |  |  |  |  |  |  |
| Grains | 87.4 | [78.5;92.9] |  | 95.5 | [88.3;98.4] |  | 95.5 | [86.5;98.6] |  | 95.5 | [70.1;99.5] |
| Pulses | 69.0 | [58.2;78] |  | 85.4 | [76.3;91.4] |  | 85.1 | [74.2;91.9] |  | 86.4 | [63.8;95.8] |
| Dairy products | 54.0 | [43.4;64.3] |  | 51.7 | [41.4;61.8] |  | 58.2 | [45.8;69.7] |  | 31.8 | [16.5;52.4] |
| Flesh foods | 4.6 | [1.7;11.8] |  | 7.9 | [3.9;15.2] |  | 10.5 | [5.2;19.8] |  | 0.0 | [0;0] |
| Egg | 3.5 | [1.1;10.4] |  | 23.6 | [15.8;33.8] |  | 25.4 | [16.1;37.6] |  | 18.2 | [6.3;42.4] |
| Green leafy vegetables | 11.5 | [6.3;20.1] |  | 33.7 | [24.6;44.2] |  | 40.3 | [29.3;52.3] |  | 13.6 | [3.9;38.1] |
| Other fruits and vegetables | 39.1 | [29.3;49.8] |  | 46.1 | [36;56.5] |  | 47.8 | [35.8;60] |  | 40.9 | [22.3;62.5] |
| Breastfeeding | 98.9 | [92;99.8] |  | 96.6 | [89.8;98.9] |  | 97.0 | [88.3;99.3] |  | 95.5 | [70.1;99.5] |
| CDD (4/7 groups) | 21.8 | [14.3;31.9] |  | 53.9 | [43.8;63.8] |  | 59.7 | [47.5;70.8] |  | 36.4 | [19.8;56.9] |
| CDD (5/8 groups) | 20.7 | [13.4;30.6] |  | 52.8 | [42.8;62.6] |  | 58.2 | [46.1;69.4] |  | 36.4 | [19.8;56.9] |
| Others |  |  |  |  |  |  |  |  |  |  |  |
| Grains | 80.5 | [74.5;85.3] |  | 94.3 | [90.2;96.8] |  | 95.3 | [90.5;97.8] |  | 91.8 | [81.1;96.7] |
| Pulses | 61.0 | [54.1;67.4] |  | 82.0 | [76.2;86.6] |  | 88.0 | [81.7;92.3] |  | 67.2 | [54.1;78.1] |
| Dairy products | 66.2 | [59.5;72.3] |  | 71.1 | [64.5;76.9] |  | 74.0 | [66.3;80.5] |  | 63.9 | [50.9;75.2] |
| Flesh foods | 2.9 | [1.3;6.3] |  | 7.6 | [4.7;12.1] |  | 9.3 | [5.6;15.2] |  | 3.3 | [0.8;12.8] |
| Egg | 3.3 | [1.6;6.9] |  | 12.8 | [9;17.9] |  | 14.7 | [9.9;21.3] |  | 8.2 | [3.5;18.2] |
| Green leafy vegetables | 8.1 | [5.1;12.7] |  | 26.5 | [21;33] |  | 32.0 | [25;40] |  | 13.1 | [6.5;24.7] |
| Other fruits and vegetables | 26.2 | [20.7;32.6] |  | 46.5 | [39.8;53.2] |  | 53.3 | [45.2;61.3] |  | 29.5 | [19.6;41.8] |
| Breastfeeding | 96.7 | [93.1;98.4] |  | 91.0 | [86.3;94.2] |  | 94.0 | [88.8;96.9] |  | 83.6 | [71.8;91.1] |
| CDD (4/7 groups) | 18.1 | [13.5;23.9] |  | 46.5 | [39.8;53.2] |  | 55.3 | [47.2;63.2] |  | 24.6 | [15.5;36.8] |
| CDD (5/8 groups) | 17.6 | [13;23.4] |  | 43.1 | [36.6;49.9] |  | 52.7 | [44.5;60.7] |  | 19.7 | [11.9;30.7] |
