# Supplementary material for: Association of BCC Module Roll-Out in SHG meetings with changes in complementary feeding and dietary diversity among children (6–23 months)? Evidence from JEEViKA in Rural Bihar, India
Source: PLoS One. 2023 Jan 5;18(1):e0279724. doi: 10.1371/journal.pone.0279724 (PMC9815627; doi:10.1371/journal.pone.0279724)
Supplement: S5 Table — (DOCX) [file pone.0279724.s008.docx]

**Supplementary Table S5:** Logistic regression results, child dietary diversity (5 out of 8 groups)

| **Background characteristics** |  | **CDD (5 out of 8 groups)** | |
| --- | --- | --- | --- |
|  |  | **Model-1** | **Model-2** |
| Household size | Less than 5 | 1 | 1 |
|  |  | [1.00,1.00] | [1.00,1.00] |
|  | 5 to 6 | 0.89 | 0.97 |
|  |  | [0.50,1.58] | [0.54,1.75] |
|  | Greater than 6 | 0.84 | 0.89 |
|  |  | [0.43,1.61] | [0.46,1.74] |
| Religion | Hindu | 1 | 1 |
|  |  | [1.00,1.00] | [1.00,1.00] |
|  | Muslim and Other | 1.17 | 1.36 |
|  |  | [0.51,2.72] | [0.58,3.19] |
| Social group | OBC and Other | 1 | 1 |
|  |  | [1.00,1.00] | [1.00,1.00] |
|  | SC/ST | 1.71* | 1.74* |
|  |  | [1.07,2.73] | [1.08,2.79] |
| Mother education | No education | 1 | 1 |
|  |  | [1.00,1.00] | [1.00,1.00] |
|  | 1 to 5 years | 0.79 | 0.76 |
|  |  | [0.39,1.59] | [0.37,1.54] |
|  | 6 to 8 years | 2.00* | 2.13* |
|  |  | [1.01,3.95] | [1.07,4.23] |
|  | More than 9 years | 2.19* | 2.39** |
|  |  | [1.14,4.20] | [1.23,4.63] |
| Husband education | No education | 1 | 1 |
|  |  | [1.00,1.00] | [1.00,1.00] |
|  | 1 to 5 years | 0.94 | 0.96 |
|  |  | [0.47,1.85] | [0.48,1.92] |
|  | 6 to 8 years | 1.74 | 1.72 |
|  |  | [0.95,3.19] | [0.94,3.17] |
|  | More than 9 years | 0.82 | 0.84 |
|  |  | [0.45,1.50] | [0.46,1.55] |
| Age of women | Less than 25 years | 1 | 1 |
|  |  | [1.00,1.00] | [1.00,1.00] |
|  | 25 to 29 years | 1.16 | 1.18 |
|  |  | [0.68,1.98] | [0.69,2.02] |
|  | More than 30 years | 1.9 | 1.85 |
|  |  | [0.92,3.93] | [0.89,3.84] |
| Occupation | Employed | 1 | 1 |
|  |  | [1.00,1.00] | [1.00,1.00] |
|  | Not employed | 0.73 | 0.74 |
|  |  | [0.44,1.21] | [0.45,1.24] |
| Sex of child | Male | 1 | 1 |
|  |  | [1.00,1.00] | [1.00,1.00] |
|  | Female | 0.87 | 0.9 |
|  |  | [0.58,1.31] | [0.59,1.36] |
| Birth order | 1 to 2 | 1 | 1 |
|  |  | [1.00,1.00] | [1.00,1.00] |
|  | 3 to 4 | 0.67 | 0.61 |
|  |  | [0.39,1.15] | [0.35,1.05] |
|  | 4+ | 0.42 | 0.38* |
|  |  | [0.17,1.02] | [0.15,0.95] |
| Wealth | Poorest | 1 | 1 |
|  |  | [1.00,1.00] | [1.00,1.00] |
|  | Poorer | 0.99 | 0.97 |
|  |  | [0.51,1.91] | [0.50,1.88] |
|  | Middle | 1.05 | 1.06 |
|  |  | [0.54,2.04] | [0.54,2.08] |
|  | Richer | 0.9 | 0.86 |
|  |  | [0.44,1.85] | [0.41,1.77] |
|  | Richest | 1.89 | 1.77 |
|  |  | [0.87,4.09] | [0.81,3.89] |
| Survey round | Pre-intervention | 1 | 1 |
|  |  | [1.00,1.00] | [1.00,1.00] |
|  | Post-intervention | 2.14** | 1.1 |
|  |  | [1.24,3.70] | [0.54,2.25] |
| Kitchen garden | No | 1 | 1 |
|  |  | [1.00,1.00] | [1.00,1.00] |
|  | Yes | 1.32 | 1.28 |
|  |  | [0.88,2.00] | [0.84,1.94] |
| Knowledge score | Low (1 to 2) | 1 | 1 |
|  |  | [1.00,1.00] | [1.00,1.00] |
|  | High (3 to 5) | 1.61 | 1.38 |
|  |  | [0.98,2.64] | [0.83,2.30] |
| Child diet preference score | Low (0 to 2) | 1 | 1 |
|  |  | [1.00,1.00] | [1.00,1.00] |
|  | Medium (3 to 5) | 1.78 | 1.56 |
|  |  | [0.67,4.75] | [0.59,4.12] |
|  | High (More than 5) | 4.01** | 2.93* |
|  |  | [1.50,10.73] | [1.07,8.05] |
| Fuel type | Wood/Agricultural | 1 | 1 |
|  |  | [1.00,1.00] | [1.00,1.00] |
|  | LPG | 2.14** | 2.14** |
|  |  | [1.32,3.44] | [1.32,3.46] |
| Age of child | 6 to 8 months | 1 | 1 |
|  |  | [1.00,1.00] | [1.00,1.00] |
|  | 9 to 11 months | 1.32 | 1.42 |
|  |  | [0.63,2.74] | [0.68,2.99] |
|  | 12 to 18 months | 2.90*** | 3.02*** |
|  |  | [1.55,5.42] | [1.60,5.69] |
|  | 19 to 23 months | 4.50*** | 4.78*** |
|  |  | [2.29,8.84] | [2.41,9.48] |
| Attended CF (session / module) | No |  | 1 |
|  |  |  | [1.00,1.00] |
|  |  |  | 2.90** |
|  | Yes |  | [1.46,5.74] |
|  | Observations | 597 | 597 |

**Note:** Model-1 based on overall sample, model-2: controlled for exposure to CF session
