# Supplementary material for: Association of BCC Module Roll-Out in SHG meetings with changes in complementary feeding and dietary diversity among children (6–23 months)? Evidence from JEEViKA in Rural Bihar, India
Source: PLoS One. 2023 Jan 5;18(1):e0279724. doi: 10.1371/journal.pone.0279724 (PMC9815627; doi:10.1371/journal.pone.0279724)
Supplement: S6 Table — (DOCX) [file pone.0279724.s009.docx]

**Supplementary Table S6:** Logistic regression results, child dietary diversity (4 out of 7 groups)

| **Background characteristics** |  | CDD (4 out of 7 groups) | |
| --- | --- | --- | --- |
|  |  | Model-1 | Model-2 |
| Household size | Less than 5 | 1 | 1 |
|  |  | [1.00,1.00] | [1.00,1.00] |
|  | 5 to 6 | 0.89 | 0.98 |
|  |  | [0.50,1.59] | [0.55,1.75] |
|  | Greater than 6 | 0.77 | 0.82 |
|  |  | [0.40,1.49] | [0.42,1.60] |
| Religion | Hindu | 1 | 1 |
|  |  | [1.00,1.00] | [1.00,1.00] |
|  | Muslim and Other | 1 | 1.15 |
|  |  | [0.43,2.32] | [0.49,2.71] |
| Social group | OBC and Other | 1 | 1 |
|  |  | [1.00,1.00] | [1.00,1.00] |
|  | SC/ST | 1.58 | 1.61* |
|  |  | [0.99,2.53] | [1.00,2.58] |
| Mother education | No education | 1 | 1 |
|  |  | [1.00,1.00] | [1.00,1.00] |
|  | 1 to 5 years | 0.79 | 0.75 |
|  |  | [0.40,1.60] | [0.37,1.53] |
|  | 6 to 8 years | 1.86 | 1.98 |
|  |  | [0.94,3.70] | [1.00,3.94] |
|  | More than 9 years | 2.41** | 2.62** |
|  |  | [1.25,4.64] | [1.35,5.07] |
| Husband education | No education | 1 | 1 |
|  |  | [1.00,1.00] | [1.00,1.00] |
|  | 1 to 5 years | 0.8 | 0.82 |
|  |  | [0.40,1.58] | [0.41,1.64] |
|  | 6 to 8 years | 1.76 | 1.74 |
|  |  | [0.96,3.21] | [0.95,3.20] |
|  | More than 9 years | 0.79 | 0.81 |
|  |  | [0.43,1.44] | [0.44,1.48] |
| Age of women | Less than 25 years | 1 | 1 |
|  |  | [1.00,1.00] | [1.00,1.00] |
|  | 25 to 29 years | 1.16 | 1.18 |
|  |  | [0.68,1.97] | [0.69,2.02] |
|  | More than 30 years | 1.7 | 1.65 |
|  |  | [0.83,3.50] | [0.80,3.43] |
| Occupation | Employed | 1 | 1 |
|  |  | [1.00,1.00] | [1.00,1.00] |
|  | Not employed | 0.74 | 0.75 |
|  |  | [0.45,1.22] | [0.45,1.25] |
| Sex of child | Male | 1 | 1 |
|  |  | [1.00,1.00] | [1.00,1.00] |
|  | Female | 0.88 | 0.9 |
|  |  | [0.58,1.32] | [0.60,1.37] |
| Birth order | 1to2 | 1 | 1 |
|  |  | [1.00,1.00] | [1.00,1.00] |
|  | 3to4 | 0.65 | 0.59 |
|  |  | [0.38,1.12] | [0.34,1.03] |
|  | 4+ | 0.48 | 0.45 |
|  |  | [0.20,1.17] | [0.18,1.10] |
| Wealth | Poorest | 1 | 1 |
|  |  | [1.00,1.00] | [1.00,1.00] |
|  | Poorer | 0.91 | 0.89 |
|  |  | [0.48,1.74] | [0.46,1.71] |
|  | Middle | 0.97 | 0.98 |
|  |  | [0.50,1.88] | [0.50,1.91] |
|  | Richer | 0.79 | 0.75 |
|  |  | [0.39,1.62] | [0.37,1.55] |
|  | Richest | 1.74 | 1.65 |
|  |  | [0.81,3.77] | [0.76,3.60] |
| Survey round | Pre-intervention | 1 | 1 |
|  |  | [1.00,1.00] | [1.00,1.00] |
|  | Post-intervention | 2.35** | 1.26 |
|  |  | [1.37,4.04] | [0.63,2.54] |
| Kitchen garden | No | 1 | 1 |
|  |  | [1.00,1.00] | [1.00,1.00] |
|  | Yes | 1.33 | 1.29 |
|  |  | [0.88,2.01] | [0.85,1.96] |
| Knowledge score | Low (1 to 2) | 1 | 1 |
|  |  | [1.00,1.00] | [1.00,1.00] |
|  | High (3 to 5) | 1.6 | 1.39 |
|  |  | [0.98,2.61] | [0.84,2.29] |
| Child diet preference score | Low (0 to 2) | 1 | 1 |
|  |  | [1.00,1.00] | [1.00,1.00] |
|  | Medium (3 to 5) | 1.32 | 1.18 |
|  |  | [0.54,3.24] | [0.48,2.87] |
|  | High (More than 5) | 2.95* | 2.18 |
|  |  | [1.20,7.29] | [0.86,5.55] |
| Cooking fuel | Wood/Agricultural | 1 | 1 |
|  |  | [1.00,1.00] | [1.00,1.00] |
|  | LPG | 2.20** | 2.21** |
|  |  | [1.37,3.55] | [1.37,3.58] |
| Age of child | 6 to 8 months | 1 | 1 |
|  |  | [1.00,1.00] | [1.00,1.00] |
|  | 9 to 11 months | 1.37 | 1.48 |
|  |  | [0.67,2.83] | [0.71,3.09] |
|  | 12 to 18 months | 2.74** | 2.84** |
|  |  | [1.47,5.09] | [1.52,5.33] |
|  | 19 to 23 months | 5.62*** | 5.96*** |
|  |  | [2.86,11.02] | [3.01,11.79] |
| Attended CF (session / module) | No |  | 1 |
|  |  |  |  |
|  | Yes |  | 2.77** |
|  |  |  |  |
|  | Observations | 597 | 597 |

**Note:** Model-1 based on overall sample, model-2: controlled for exposure to CF session
