# Supplementary material for: Association of BCC Module Roll-Out in SHG meetings with changes in complementary feeding and dietary diversity among children (6–23 months)? Evidence from JEEViKA in Rural Bihar, India
Source: PLoS One. 2023 Jan 5;18(1):e0279724. doi: 10.1371/journal.pone.0279724 (PMC9815627; doi:10.1371/journal.pone.0279724)
Supplement: S7 Table — (DOCX) [file pone.0279724.s010.docx]

**Supplementary Table S7:** Logistic regression results, child dietary diversity excluding knowledge and preference score

| **Background characteristics** |  | **CDD (4 out of 7 groups)** | | **CDD (5 out of 8 groups)** | |
| --- | --- | --- | --- | --- | --- |
|  |  | **Model-1** | **Model-2** | **Model-1** | **Model-2** |
| Household size | Less than 5 | 1 | 1 | 1 | 1 |
|  |  | [1.00,1.00] | [1.00,1.00] | [1.00,1.00] | [1.00,1.00] |
|  | 5 to 6 | 0.83 | 0.97 | 0.82 | 0.96 |
|  |  | [0.47,1.45] | [0.54,1.72] | [0.47,1.43] | [0.54,1.71] |
|  | Greater than 6 | 0.75 | 0.83 | 0.81 | 0.9 |
|  |  | [0.40,1.44] | [0.43,1.61] | [0.43,1.54] | [0.47,1.74] |
| Religion | Hindu | 1 | 1 | 1 | 1 |
|  |  | [1.00,1.00] | [1.00,1.00] | [1.00,1.00] | [1.00,1.00] |
|  | Muslim and Other | 1.07 | 1.26 | 1.24 | 1.48 |
|  |  | [0.47,2.42] | [0.55,2.91] | [0.55,2.80] | [0.64,3.40] |
| Social group | OBC and Other | 1 | 1 | 1 | 1 |
|  |  | [1.00,1.00] | [1.00,1.00] | [1.00,1.00] | [1.00,1.00] |
|  | SC/ST | 1.72* | 1.72* | 1.85** | 1.85** |
|  |  | [1.09,2.72] | [1.08,2.74] | [1.17,2.93] | [1.16,2.96] |
| Mother education | No education | 1 | 1 | 1 | 1 |
|  |  | [1.00,1.00] | [1.00,1.00] | [1.00,1.00] | [1.00,1.00] |
|  | 1 to 5 years | 0.88 | 0.78 | 0.89 | 0.79 |
|  |  | [0.44,1.73] | [0.39,1.58] | [0.45,1.76] | [0.39,1.61] |
|  | 6 to 8 years | 2.14* | 2.21* | 2.33* | 2.41* |
|  |  | [1.10,4.17] | [1.13,4.34] | [1.20,4.51] | [1.23,4.71] |
|  | More than 9 years | 2.63** | 2.83** | 2.41** | 2.60** |
|  |  | [1.39,4.98] | [1.47,5.45] | [1.28,4.55] | [1.36,5.00] |
| Husband education | No education | 1 | 1 | 1 | 1 |
|  |  | [1.00,1.00] | [1.00,1.00] | [1.00,1.00] | [1.00,1.00] |
|  | 1 to 5 years | 0.83 | 0.85 | 0.97 | 1 |
|  |  | [0.42,1.62] | [0.42,1.69] | [0.50,1.89] | [0.50,1.98] |
|  | 6 to 8 years | 1.90* | 1.83* | 1.90* | 1.83 |
|  |  | [1.05,3.42] | [1.00,3.34] | [1.05,3.43] | [1.00,3.35] |
|  | More than 9 years | 0.9 | 0.88 | 0.94 | 0.93 |
|  |  | [0.50,1.63] | [0.48,1.62] | [0.52,1.70] | [0.51,1.70] |
| Age of women | Less than 25 years | 1 | 1 | 1 | 1 |
|  |  | [1.00,1.00] | [1.00,1.00] | [1.00,1.00] | [1.00,1.00] |
|  | 25 to 29 years | 1.16 | 1.18 | 1.18 | 1.19 |
|  |  | [0.69,1.96] | [0.69,2.02] | [0.70,1.98] | [0.70,2.04] |
|  | More than 30 years | 1.74 | 1.68 | 1.98 | 1.9 |
|  |  | [0.86,3.54] | [0.81,3.46] | [0.97,4.03] | [0.92,3.93] |
| Occupation | Employed | 1 | 1 | 1 | 1 |
|  |  | [1.00,1.00] | [1.00,1.00] | [1.00,1.00] | [1.00,1.00] |
|  | Not employed | 0.72 | 0.74 | 0.71 | 0.73 |
|  |  | [0.44,1.19] | [0.44,1.22] | [0.44,1.17] | [0.44,1.21] |
| Sex of child | Male | 1 | 1 | 1 | 1 |
|  |  | [1.00,1.00] | [1.00,1.00] | [1.00,1.00] | [1.00,1.00] |
|  | Female | 0.94 | 0.96 | 0.94 | 0.96 |
|  |  | [0.63,1.40] | [0.64,1.44] | [0.63,1.41] | [0.64,1.44] |
| Birth order | 1 to 2 | 1 | 1 | 1 | 1 |
|  |  | [1.00,1.00] | [1.00,1.00] | [1.00,1.00] | [1.00,1.00] |
|  | 3 to 4 | 0.73 | 0.62 | 0.75 | 0.64 |
|  |  | [0.43,1.24] | [0.36,1.07] | [0.44,1.28] | [0.37,1.10] |
|  | 4+ | 0.52 | 0.45 | 0.45 | 0.38* |
|  |  | [0.22,1.23] | [0.18,1.09] | [0.19,1.07] | [0.16,0.95] |
| Wealth | Poorest | 1 | 1 | 1 | 1 |
|  |  | [1.00,1.00] | [1.00,1.00] | [1.00,1.00] | [1.00,1.00] |
|  | Poorer | 0.81 | 0.82 | 0.88 | 0.89 |
|  |  | [0.43,1.53] | [0.43,1.57] | [0.46,1.67] | [0.47,1.72] |
|  | Middle | 0.92 | 0.94 | 0.99 | 1.02 |
|  |  | [0.48,1.77] | [0.48,1.83] | [0.51,1.91] | [0.52,1.99] |
|  | Richer | 0.7 | 0.7 | 0.79 | 0.79 |
|  |  | [0.35,1.40] | [0.34,1.41] | [0.39,1.59] | [0.39,1.61] |
|  | Richest | 1.61 | 1.54 | 1.74 | 1.66 |
|  |  | [0.76,3.43] | [0.71,3.33] | [0.82,3.70] | [0.77,3.60] |
| Survey round | Pre-intervention | 1 | 1 | 1 | 1 |
|  |  | [1.00,1.00] | [1.00,1.00] | [1.00,1.00] | [1.00,1.00] |
|  | Post-intervention | 4.59*** | 1.66 | 4.27*** | 1.45 |
|  |  | [3.00,7.01] | [0.87,3.17] | [2.79,6.53] | [0.75,2.80] |
| Kitchen garden | No | 1 | 1 | 1 | 1 |
|  |  | [1.00,1.00] | [1.00,1.00] | [1.00,1.00] | [1.00,1.00] |
|  | Yes | 1.46 | 1.35 | 1.46 | 1.34 |
|  |  | [0.98,2.19] | [0.89,2.03] | [0.98,2.19] | [0.89,2.02] |
| Fuel type | Wood/Agricultural | 1 | 1 | 1 | 1 |
|  |  | [1.00,1.00] | [1.00,1.00] | [1.00,1.00] | [1.00,1.00] |
|  | LPG | 2.39*** | 2.34*** | 2.34*** | 2.28*** |
|  |  | [1.50,3.82] | [1.45,3.76] | [1.47,3.74] | [1.42,3.67] |
| Age of child | 6 to 8 months | 1 | 1 | 1 | 1 |
|  |  | [1.00,1.00] | [1.00,1.00] | [1.00,1.00] | [1.00,1.00] |
|  | 9 to 11 months | 1.39 | 1.53 | 1.32 | 1.45 |
|  |  | [0.68,2.84] | [0.74,3.17] | [0.64,2.71] | [0.69,3.04] |
|  | 12 to 18 months | 2.90*** | 2.98*** | 3.07*** | 3.16*** |
|  |  | [1.57,5.36] | [1.60,5.57] | [1.65,5.69] | [1.68,5.94] |
|  | 19 to 23 months | 5.58*** | 5.99*** | 4.46*** | 4.77*** |
|  |  | [2.87,10.87] | [3.03,11.82] | [2.29,8.68] | [2.41,9.44] |
| Attended CF (session / module) | No | - | 1 | - | 1 |
|  |  | - | - [1.00,1.00] | | [1.00,1.00] |
|  | Yes | - | 3.81*** | - | 4.12*** |
|  |  | - | - [2.03,7.15] | | [2.17,7.81] |
|  | Observations | 597 | 597 | 597 | 597 |
