# Supplementary material for: Association of BCC Module Roll-Out in SHG meetings with changes in complementary feeding and dietary diversity among children (6–23 months)? Evidence from JEEViKA in Rural Bihar, India
Source: PLoS One. 2023 Jan 5;18(1):e0279724. doi: 10.1371/journal.pone.0279724 (PMC9815627; doi:10.1371/journal.pone.0279724)
Supplement: S8 Table — (DOCX) [file pone.0279724.s011.docx]

**Supplementary Table S8:** Sensitivity analysis based on alternative estimators for the ATT effects for child dietary diversity

|  | Unmatched |  | % reduction | t test |  | V(T) / V(C) |
| --- | --- | --- | --- | --- | --- | --- |
| Variable | Matched | %bias | \|bias\| | t | p>t |  |
|  |  |  |  |  |  |  |
| 1.hhsize | U | -8.8 |  | -0.98 | 0.327 | . |
|  | M | 0.9 | 89.3 | 0.1 | 0.921 | . |
|  |  |  |  |  |  |  |
| 2.hhsize | U | -5 |  | -0.56 | 0.573 | . |
|  | M | -1.9 | 62.2 | -0.2 | 0.842 | . |
|  |  |  |  |  |  |  |
| 1.religion | U | -3 |  | -0.34 | 0.735 | . |
|  | M | -3.8 | -24 | -0.39 | 0.697 | . |
|  |  |  |  |  |  |  |
| 1.caste | U | 3.4 |  | 0.39 | 0.7 | . |
|  | M | -1 | 70.9 | -0.1 | 0.918 | . |
|  |  |  |  |  |  |  |
| 1.womanedu | U | 8.8 |  | 0.99 | 0.322 | . |
|  | M | -4.3 | 50.7 | -0.42 | 0.675 | . |
|  |  |  |  |  |  |  |
| 2.womanedu | U | -1.2 |  | -0.14 | 0.891 | . |
|  | M | -11.6 | -850.4 | -1.14 | 0.253 | . |
|  |  |  |  |  |  |  |
| 3.womanedu | U | 14.7 |  | 1.66 | 0.098 | . |
|  | M | 11.5 | 21.5 | 1.18 | 0.237 | . |
|  |  |  |  |  |  |  |
| 1.husbedu | U | 1 |  | 0.11 | 0.913 | . |
|  | M | -14 | -1334.8 | -1.35 | 0.177 | . |
|  |  |  |  |  |  |  |
| 2.husbedu | U | -6.8 |  | -0.76 | 0.45 | . |
|  | M | -2.4 | 64.6 | -0.25 | 0.799 | . |
|  |  |  |  |  |  |  |
| 3.husbedu | U | 4.4 |  | 0.5 | 0.62 | . |
|  | M | 8.1 | -83.6 | 0.85 | 0.394 | . |
|  |  |  |  |  |  |  |
| 1.womanage | U | -10 |  | -1.12 | 0.264 | . |
|  | M | -15.1 | -51 | -1.57 | 0.118 | . |
|  |  |  |  |  |  |  |
| 2.womanage | U | -11.8 |  | -1.32 | 0.188 | . |
|  | M | 12.3 | -3.7 | 1.41 | 0.159 | . |
|  |  |  |  |  |  |  |
| 1.womanempl | U | -16.7 |  | -1.88 | 0.061 | . |
|  | M | 4.3 | 74.1 | 0.42 | 0.672 | . |
|  |  |  |  |  |  |  |
| 1.gender | U | 17.7 |  | 1.99 | 0.047 | . |
|  | M | -8.3 | 53 | -0.86 | 0.388 | . |
|  |  |  |  |  |  |  |
| 1.parity1 | U | -10.6 |  | -1.19 | 0.235 | . |
|  | M | -12.9 | -21.5 | -1.34 | 0.18 | . |
|  |  |  |  |  |  |  |
| 2.parity1 | U | -10.1 |  | -1.13 | 0.261 | . |
|  | M | 13.6 | -34.4 | 1.61 | 0.107 | . |
|  |  |  |  |  |  |  |
| 1.wealth | U | -0.1 |  | -0.01 | 0.989 | . |
|  | M | -11.5 | -9181.2 | -1.16 | 0.248 | . |
|  |  |  |  |  |  |  |
| 2.wealth | U | -3 |  | -0.33 | 0.741 | . |
|  | M | -4.6 | -55.7 | -0.48 | 0.634 | . |
|  |  |  |  |  |  |  |
| 3.wealth | U | -0.4 |  | -0.05 | 0.961 | . |
|  | M | 9.3 | -2021.4 | 1.01 | 0.313 | . |
|  |  |  |  |  |  |  |
| 4.wealth | U | 4.4 |  | 0.5 | 0.62 | . |
|  | M | 1.1 | 74.3 | 0.12 | 0.907 | . |
|  |  |  |  |  |  |  |
| 1.homegarden | U | 15.3 |  | 1.72 | 0.086 | . |
|  | M | 7.4 | 51.9 | 0.77 | 0.443 | . |
|  |  |  |  |  |  |  |
| 1.ntfuel | U | -7.9 |  | -0.88 | 0.378 | . |
|  | M | 0 | 100 | 0 | 1 | . |

Note: * if variance ratio outside [0.77; 1.31] for U and [0.77; 1.31] for M

| Sample | Pseudo-R^2^ | LR-Chi^2^ | p>chi^2^ | Mean Bias | B | R | %Var |
| --- | --- | --- | --- | --- | --- | --- | --- |
| Unmatched | 0.038 | 26.39 | 0.235 | 7.5 | 46.4* | 1.11 | . |
| Matched | 0.024 | 14.25 | 0.892 | 7.3 | 36.6* | 0.85 | . |

* if B>25%, R outside [0.5; 2]
