# Supplementary material for: Association of BCC Module Roll-Out in SHG meetings with changes in complementary feeding and dietary diversity among children (6–23 months)? Evidence from JEEViKA in Rural Bihar, India
Source: PLoS One. 2023 Jan 5;18(1):e0279724. doi: 10.1371/journal.pone.0279724 (PMC9815627; doi:10.1371/journal.pone.0279724)
Supplement: S9 Table — (DOCX) [file pone.0279724.s012.docx]

**Supplementary Table S9:** Sensitivity analysis based on alternative estimators for the ATT effects for child dietary diversity

| Estimation technique | ATT estimates  CDD (4/7 groups) | ATT estimates  CDD (5/8 groups) |
| --- | --- | --- |
| Propensity score matching (caliper 0.1) | 0.294*** | 0.281*** |
|  | [0.184; 0.404] | [0.173; 0.389] |
| Regression adjustment | 0.381*** | 0.366*** |
|  | [0.300; 0.462] | [0.285; 0.446] |
| Inverse probability weighting | 0.379*** | 0.363*** |
|  | [0.297; 0.460] | [0.282; 0.444] |
| IPW and RA matching | 0.379*** | 0.363*** |
|  | [0.297; 0.460] | [0.282; 0.444] |
| Nearest neighbour matching | 0.357 *** | 0.343 *** |
|  | [0.261; 0.452] | [0.247; 0.439] |
| Nearest neighbour matching (bias adjusted) | 0.340*** | 0.327*** |
|  | [0.244; 0.436] | [0.230; 0.423] |
| Propensity score matching (neighbours 2) | 0.323*** | 0.358*** |
|  | [0.240; 0.407] | [0.263; 0.453] |

Note: Overidentification test for covariate balance cannot be rejected (p > chi^2^ = 0.995).
