# Supplementary material for: Association of BCC Module Roll-Out in SHG meetings with changes in complementary feeding and dietary diversity among children (6–23 months)? Evidence from JEEViKA in Rural Bihar, India
Source: PLoS One. 2023 Jan 5;18(1):e0279724. doi: 10.1371/journal.pone.0279724 (PMC9815627; doi:10.1371/journal.pone.0279724)
Supplement: S1 Text — (DOCX) [file pone.0279724.s013.docx]

**Supplementary Text: Propensity Score Matching Note**

The PSM approach has gained popularity as an effective tool to reduce selection bias that may crop in because of unobserved differences in demographic and socioeconomic characteristics between control and treatment groups. The PSM method comprises of two key steps as follows: First, a logit regression is used to identify the socioeconomic characteristics of the sample households and use this information to develop propensity scores (defined by Rosenbaum and Rubin (1983) as the conditional probability of treatment given pre-treatment features) [42]. Thereafter, the comparison and treatment households are matched based on comparisons of their propensity scores. Respondents which are having similar propensity scores are considered more comparable than those who are deviant in terms of their propensity scores. The variables used to generate propensity score based on the matching algorithm are as follows: sex and birth order of the child, household size, social group, religion, age, education and employment status of mother, education of father, household wealth index quintile, availability of kitchen garden and type of cooking fuel.

The PSM algorithm yields two parameters of interest viz. the average treatment effect (ATE) and average treatment effect on treated (ATT). The ATE measures the mean impact on respondents who were exposed and is technically defined as follows:

ATE = E (δ) = E (Y1– Y0)

Where E (δ) represents the mean average and Y1 represents the potential outcome describing the dietary diversity status of children of the exposed respondent and Y0 describing the expected outcome of the comparison group respondents.

In the post-treatment case, the counterfactual model helps to understand the difference between the average outcome of those who received the treatment and the unobserved counterfactual. Similarly, the ATT is estimated as follows:

ATT = E(Y1|D = 1) - E(Y0|D = 1)

Where E(Y1|D = 1) is the average outcome of the exposed respondent and E(Y0|D = 1) is the estimate for the counterfactual.

The ATE and ATT estimates are computed using the *teffects psmatch* routine in Stata 16.0. The propensity score matching estimates are based on caliper setting of 0.1 i.e. the maximum distance imposed for which two observations are potential neighbors. We perform various covariate balancing tests to confirm common support for the propensity scores for the treatment and comparison groups. The variance ratios, overlap and box plot for the matched sample are also performed to test the robustness of propensity score matching analysis. We also present the ATE and ATT estimates based on the nearest neighbor matching technique. For a sensitivity analysis, ATT values based on alternative estimation techniques are presented in Supplementary Table S5.
